# Integrated Molecular Modeling and Machine Learning for Drug Design

**DOI:** 10.1021/acs.jctc.3c00814

**Published:** 2023-10-26

**Authors:** Song Xia, Eric Chen, Yingkai Zhang

**Affiliations:** † Department of Chemistry, New York University, New York, New York 10003, United States; ‡ Simons Center for Computational Physical Chemistry at New York University, New York, New York 10003, United States; § NYU-ECNU Center for Computational Chemistry at NYU Shanghai, Shanghai 200062, China

## Abstract

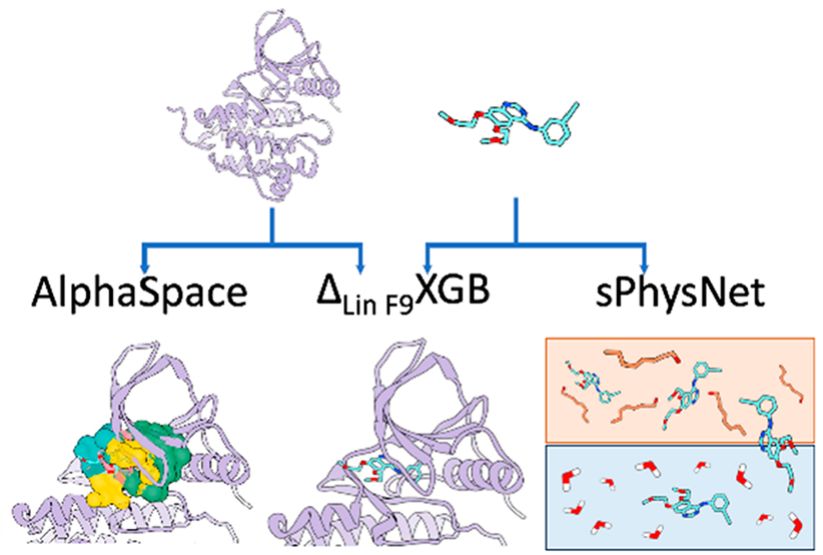

Modern therapeutic development often involves several stages that are interconnected, and multiple iterations are usually required to bring a new drug to the market. Computational approaches have increasingly become an indispensable part of helping reduce the time and cost of the research and development of new drugs. In this Perspective, we summarize our recent efforts on integrating molecular modeling and machine learning to develop computational tools for modulator design, including a pocket-guided rational design approach based on AlphaSpace to target protein–protein interactions, delta machine learning scoring functions for protein–ligand docking as well as virtual screening, and state-of-the-art deep learning models to predict calculated and experimental molecular properties based on molecular mechanics optimized geometries. Meanwhile, we discuss remaining challenges and promising directions for further development and use a retrospective example of FDA approved kinase inhibitor Erlotinib to demonstrate the use of these newly developed computational tools.

## Introduction

The cost of research and development of new drugs has risen over the past decade.^1−3^ There is great interest in advancing computational methods, including molecular modeling and machine learning (ML), to greatly accelerate the process of drug discovery and development.^4−10^ The goal of drug discovery is to find small molecules that inhibit the disease-related target and, therefore, modulate the disease. Meanwhile, the pharmacokinetic properties including absorption, distribution, metabolism, excretion, and toxicity (ADME/T) of small molecules also need to be considered in the drug development process.^11^ The success of drug development requires both good efficacy and the desired pharmacokinetic properties of the drug molecules. Computer-aided drug design (CADD) offers valuable tools in finding candidate compounds in all stages of drug discovery and development.

**Figure 1:**
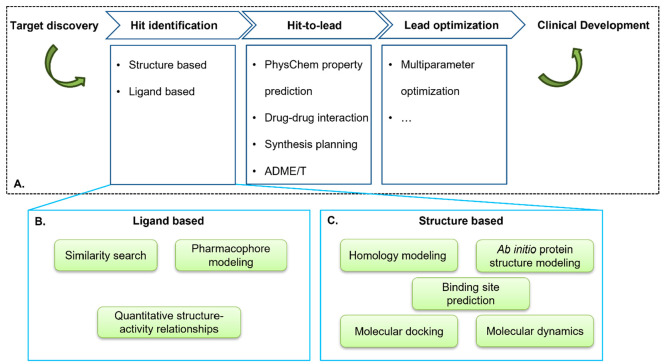
Roles of computational models at the stages of drug discovery and development.

It should be noted that a modern therapeutic development process often involves multiple nonlinearly interconnected stages.^12^Figure 1A shows a simplified “pipeline” of therapeutic development where CADD tools are playing important roles. Hit identification is the first step which involves finding the compounds with desired bioactivity.^13^ Ligand based methods use prior knowledge to predict new drug compounds with similar bioactivities (Figure 1B).^14^ Similarity search,^15^ pharmacophore modeling,^16,17^ and quantitative structure–activity relationships^18,19^ are common tools for ligand based drug screening. Structure based methods such as molecular docking^20−26^ and molecular dynamics simulations^27−32^ are common computational tools used to predict the binding affinity between the protein and ligand (Figure 1C). Binding site prediction tools^33−39^ offer information on the protein surface where a ligand molecule binds to produce the desired output (activation, inhibition, or modulation).^5^ If the structure of the protein is not solved experimentally, homology modeling^40−43^ and *ab initio* protein structure modeling^44−47^ approaches can be used to predict the protein structure.

After discovering the hit compounds, subsequent steps involve producing more potent and selective compounds with desirable physicochemical properties.^13^ It is estimated that over 40% of drug candidates are withdrawn in preclinical tests due to ADME/T concerns.^48^ To address the problem, a number of computational models have been developed to predict the ADME/T properties of small molecules.^49−60^ The accurate prediction of molecular properties requires models to learn more robust molecular representations for 1D SMILES,^61^ 2D graph,^62^ and 3D geometry.^63^

As computational tools at all stages of drug discovery and development have been extensively reviewed recently,^64,65^ in this perspective, we focus on the recent progress related to our group. Specifically, we first summarize protein pocket identification and analysis methods based on fragment-centric topographical mapping. Subsequently, we discuss recent advances and promising directions in developing ML and deep learning (DL)-based molecular docking models. Finally, we describe our recent efforts to improve small molecule representation learning and explore chemical space^66^ more efficiently.

## 1 Protein Pocket Identification and Representation

### 1.1 Protein Pocket Identification: Fragment-Centric Topographical Mapping

In contemporary approaches to structure-based drug design, focus is placed on the analysis of concave regions found on biomolecular surfaces. These regions can vary from well-defined small-molecule binding sites to extensive protein–protein interaction (PPI) interfaces.^67^ Gaining a deeper understanding of concave biomolecular surfaces holds immense potential for various applications in modulator design, such as lead optimization, enhanced ligand screening, and even the identification of previously undiscovered druggable sites.^67−69^ Furthermore, comparisons of these surfaces investigate ligand selectivity, off-target effects, and polypharmacological activity.^70,71^

One of the main tasks in analyzing the protein surfaces is identifying binding sites. There are a number of algorithms that do this, and these can be broadly classified as geometry-^35,37,72−76^ or energy-based methods.^68,77−81^ Geometry-based methods map out the concavities on the surface using grids or alpha-spheres, while energy-based methods calculate the interaction energy or surface accessibility between the protein and diverse small-molecule probes. The next step usually requires ML methods to characterize the binding site. For example, some methods use clustering to agglomerate data points into binding pockets and others use regression to score the concavities on the protein surface for their ligandability.^82^ Most methods can correctly predict and rank the voluminous and well-defined ligand binding pockets in *holo* structures.^72,83,84^

**Figure 2:**
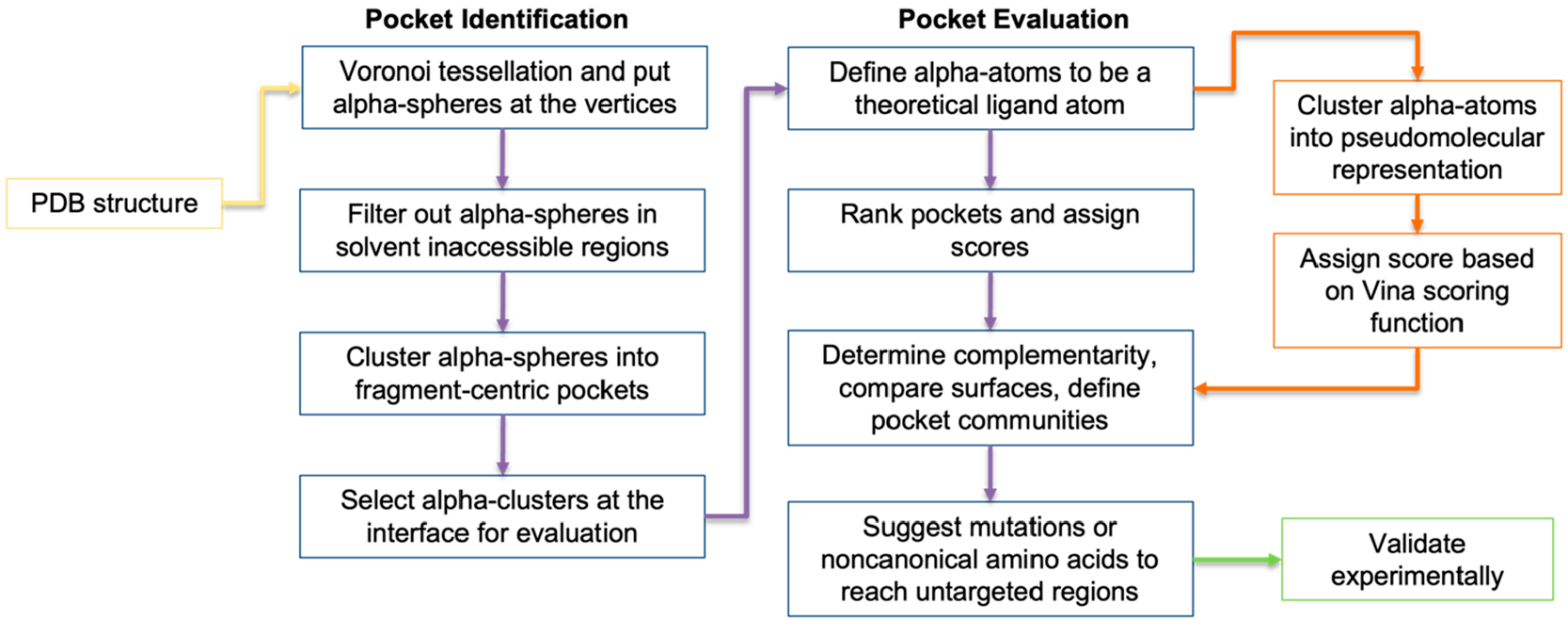
Workflow of AlphaSpace to optimize a ligand to bind to a protein begins with a selected PDB structure and follows the purple arrows until the optimization is validated experimentally. The additions introduced by AlphaSpace2.0 follow the orange arrows.

The alpha-sphere geometry-based representation of the concavities is an established strategy that relies on Voronoi diagrams.^34,35,73,74,85−87^ Voronoi diagrams form polyhedra defined by the planes that bisect adjacent atoms, resulting in a tessellation of the concave region. Alpha-spheres are placed at the vertices of the Voronoi diagram. These alpha-spheres are then clustered to represent binding pockets. Rooklin et al. developed a python program named AlphaSpace, an alpha-sphere-based method developing on the popular method fpocket,^35^ that leverages concepts of fragment-based drug discovery and caters to the analysis of PPI (Figure 2).^34^ To this aim, AlphaSpace uses a tuned clustering method to demarcate pockets that reflect on average one fragment per pocket and defines alpha-atoms, a theoretical ligand atom whose properties reflect a docked fragment. It represents entire PPI surfaces by retaining shallower fragment-centric pockets during the evaluation stage and suggests targetable pockets near the binding partner for ligand optimization. Furthermore, Katigbak et al. developed AlphaSpace 2.0, a python package that clusters the alpha-atoms to a β-cluster, a pseudomolecular representation of the concave site comprised of β-atoms (Figure 2).^33^ Each β-atom is replaced with a probe atom that iterates through different Vina atom types. The AutoDock Vina score is calculated for each atom type of each probe atom. Then the best Vina score for each probe atom is summed to generate the overall β-score. This β-score reflects the maximum theoretical docking score of the β-cluster.

Both AlphaSpace and AlphaSpace2 offer alignment-free and alignment-based pocket matching protocols, respectively, allowing for comparisons of binding sites between structures within the alpha-sphere framework.^33,34^ Both methods assess the change in the inhibitor binding site of PPI interfaces using the curated 2P2I database, which provides structures of a protein in the *apo*, PPI complex, or inhibitor bound PPI complex states. In both cases they can observe the binding pockets on the apo structure that corresponds to the inhibitor binding site, and AlphaSpace2 reports similar ligand-ability estimates of that binding site across structures. However, on the CryptoSite database consisting of structures where a pocket forms in the *holo* state but not the *apo* state, AlphaSpace2 reports differences in ligand-ability estimates.

DL methods are also gaining prominence due to their ability to learn complex relationships from raw protein data and predict binding surfaces.^82,88^ DL architectures can learn the representation and functional roles of the binding pockets without first preprocessing the data to explicitly detect the pockets. Mylonas et al. developed DeepSurf, a 3D-convolutional neural network (CNN) trained on voxels placed on the protein surface.^89^ It had superior performance compared to other methods tested in the SHREC 2022 contest at identifying druggable sites on diverse data sets of *apo* and *holo* structures.^83,89^ Gainza et al. proposed MaSIF, a surface representation framework that uses overlapping radial patches characterized by a chemical and geometric fingerprint.^90,91^ These patches can be input into geometric neural network architectures and trained to perform particular tasks, including predicting PPI and ligand sites. Most recently, Smith et al. developed Graph Attention Site Prediction (GrASP) which uses a graph attention network (GAT) architecture to encode the binding site from a protein structure graph. This model is trained on a modified version of sc-PDB to predict the likelihood of an atom to be part of the binding site. These atoms are then clustered and aggregated into binding sites with ligand-ability scores.^92^ This results in superior precision and recall compared to another graph neural network (GNN)-based method, P2Rank, on the COACH420 and HOLO4K benchmarks.^92^

### 1.2 Experimentally Validating Predictions of Underutilized but Targetable Regions

#### AlphaSpace has guided the optimization of minimal protein mimetics that target PPIs by expanding mimetics to target secondary binding sites and incorporating noncanonical amino acids to reach untargeted binding pockets

(93−95) Guided by the binding pocket analysis, researchers introduced a terminal tryptophan of a p53 mimetic to interact with a previously untargeted secondary site which resulted in an ∼10-fold improvement of binding affinity to MdmX and Mdm2.^95^ This interaction was verified to bind to the secondary site through NMR. In another case, optimization steps were able to reverse the loss of binding affinity resulting from truncating the full length protein to a minimal mimetic. The truncated helical MML-mimetic bound poorly to the KIX domain of the p300/CBP coactivator (*K*_d_: >100 μm), but a MML mimetic mutated with noncanonical amino acids was optimized to a similar binding affinity (*K*_d_: 3.3 ± 0.5 μm) as the full-length wildtype MML (*K*_d_: 1.0 ± 0.3 μm).^93^ Moreover, suggested mutations to the cross-linked helix dimers of the NEMO coiled coil reversed the binding affinity loss due to the aforementioned. These mimetics were also able to inhibit the native PPI *in vivo*.^94^ In these cases, the role of AlphaSpace was to suggest initial fragments for improvement, but expertise and further experimental optimization were necessary to generate stable mimetics and maximize protein–ligand binding affinity.^93−96^

AlphaSpace has also guided the analysis of protein structures for targetable binding sites to design small molecule inhibitors and understand their binding mechanisms. Some computational experiments qualitatively studied the interaction mechanisms and binding modes by docking inhibitors to the most targetable pockets of the transmembrane protein ABCG2 and by running molecular dynamics (MD) simulations of the complexes.^97,98^ Other binding pocket analyses led to successful prospective docking studies. Hou et al. detected a cryptic pocket in a metastable state of the *apo* striatal-enriched protein tyrosine phosphatase (STEP) from MD simulations. Ensemble docking to this state at the cryptic pocket resulted in 11 novel inhibitors (IC_50_ = 9.7–47.7 μm).^99^ These experimental validations highlight the utility and role that AlphaSpace can play in identifying pockets to develop ligands with improved binding.

## 2 Protein–Ligand Docking in the Machine-Learning Era

Employing computational methods to discover bioactive compounds is becoming an indispensable technique to accelerate the process of drug discovery,^100−107^ leading to the successful discovery of several approved drugs.^104,108−110^ The drug discovery process encompasses two major categories: ligand-based drug discovery (LBDD) and structure-based drug discovery (SBDD). LBDD is an indirect approach that employs quantitative structure–activity relationship methods, molecular similarity, and pharmacophore modeling to predict various properties of compounds.^14^ On the other hand, SBDD involves using the three-dimensional structures of the protein and small molecule to identify or optimize drug compounds.^101,111^ SBDD focuses on recognizing binding sites and essential interactions crucial to the biological functions of the target. By doing so, it suggests therapeutic compounds that can compete with these crucial interactions, thereby disrupting biological pathways effectively.

Virtual screening (VS) is a method within SBDD where extensive libraries of commercially available, druglike chemical compounds are computationally screened against target proteins with known or predicted 3D structures in a high-throughput manner. The primary goal of VS is to identify potential compounds that exhibit a high predicted binding affinity for the target protein. These selected compounds are then subjected to experimental testing to validate their actual binding capabilities.^112,113^

Molecular docking is a widely used computational procedure in VS protocols.^114−117^ It operates based on the lock-and-key^118^ model of drug action and predicts the noncovalent interactions between the target and small molecules. In structure-based VS, molecular docking methods are commonly used to assess large libraries of molecules.^26^

### 2.1 Protein–Ligand Scoring Functions

Molecular docking programs rely on a scoring function to evaluate the binding affinity and optimize the binding orientation between protein and ligand.^21−23,25,119−121^ The scoring function plays a pivotal role in accurately predicting how well the ligand binds to the target protein. Consequently, developing a robust, efficient, and accurate scoring function is a critical component of molecular docking.^107^

A rigorous theoretical prediction of protein–ligand binding affinity requires the calculation of the free energy difference between the bound and unbound state. However, existing methods for free energy calculation, such as free energy perturbation^122,123^ and thermodynamic integration,^124,125^ are computationally expensive, making them impractical for virtual screening over a large set of compounds. To overcome this limitation, scoring functions serve as fast and approximate computational methods to evaluate the strength of noncovalent interactions between proteins and ligands, based on their 3D binding poses.^126−128^ They are widely used in SBDD to determine the binding mode of ligands (docking power), predict the binding affinity between proteins and ligands (scoring power), identify hits from a large compound library for given protein targets (screening power), and perform structure–activity relationship analyses for hit-to-lead and lead optimization.^105,129,130^ It is important to note that scoring functions provide estimations of ligand binding affinity, and these predicted scores may not align closely with binding affinities determined through rigorous methods, such as free energy perturbation. For an in-depth understanding of terms such as “scoring power”, “docking power”, and “screening power”, we direct readers to the comprehensive definitions provided in the paper “Comparative Assessment of Scoring Functions: The CASF-2016 Update”.^131^

Classical scoring functions use a linear combination of force-field or interaction descriptors to evaluate the binding affinity between proteins and ligands. They can be categorized into different types based on their underlying principles. Physics-based scoring functions use the fundamental molecular physics terms derived from experimental data and/or QM calculations;^21,119,121^ knowledge-based scoring functions use the statistical potentials derived from experimentally determined protein–ligand structures;^132−135^ and empirical scoring functions make predictions by fitting experimentally measured binding affinity data using linear regression on a set of physical descriptors.^21−23,120,136−139^

In recent years, ML-based scoring functions have garnered increasing attention due to the rapid growth of experimental data and advancements in computational infrastructure.^140,141^ Unlike classical scoring functions that adopt linear forms, ML-based scoring functions have the capability to learn more complex functional forms using ML algorithms such as support vector machine (SVM),^142^ random forest (RF),^143^ and extreme gradient boosting (XGB).^144^ In 2010, Ballester et al. introduced RF-score which is the first ML-based scoring function that outperforms classical scoring functions in terms of scoring power.^145^ In 2013, Zillian et al. developed SFCscore^RF^ which achieved significantly higher scoring power on common benchmarks.^146^ However, both models performed much worse when performing docking and screening tasks compared to classical scoring functions.^147,148^ The reader is directed to this review by Chao et al.^107^ for more in-depth discussion on scoring functions.

#### The Δ-ML strategy improves the scoring-ranking-docking-screening power of ML-based scoring functions

To address this problem, Wang et al. applied a Δ-ML strategy, which involves using ML to predict the correction between experimental binding affinity and computationally predicted binding affinity. The final predicted score is obtained by adding this ML correction to classical scoring functions. The first model to employ this strategy is Δ_Vina_RF_20_,^149^ a random forest model that predicts the corrections to the Vina score. It uses pharmacophore-based solvent-accessible surface area descriptors and AutoDock Vina features as input. Δ_Vina_RF_20_ demonstrated superior performance in all power tests on both CASF-2013 and CASF-2007 benchmarks compared to classical scoring functions.^150,151^ Subsequently, Lu et al. have proposed the Δ_Vina_XGB model which uses XGB trees.^152,153^ This model incorporates features from explicit water molecules, metal ions, and ligand conformational stability in addition to the Δ_Vina_RF_20_ features.^152^ The Δ_Vina_XGB model outperformed other scoring functions on the CASF-2016 benchmark data set.^131^ Recently, Yang et al. have developed the Δ_Lin___F9_XGB model^154^ based on a classical scoring function named Lin_F9.^139^ Lin_F9 is a linear empirical scoring function that builds on AutoDock Vina, overcoming some of Vina’s limitations by introducing new empirical terms for better scoring accuracy. By applying the Δ-ML strategy to the Lin_F9 scoring function, and improving feature selection and the training set, Δ_Lin___F9_XGB achieves superior scoring, ranking, and screening performance on the CASF-2016 benchmark.^154^

In addition to the CASF-2016 benchmark data set, previous models have undergone extensive testing on the LIT-PCBA data set, specifically designed for virtual screening and ML purposes.^155^ Preliminary results have shown that the LIT-PCBA data set is challenging due to the high imbalance of active/inactive compounds, common molecular properties shared between active and inactive compounds, and weak potencies of the active compounds.^154,156^ The average top 1% enrichment factor achieved by Δ_Lin___F9_XGB is 5.55, which outperforms other scoring methods such as AutoDock Vina, Δ_Vina_RF_20_, and Lin_F9.

#### Learning distance likelihood potential boosts the docking and screening performance of DL-based scoring functions

Aside from traditional ML-based scoring functions, the emergence of DL methods has introduced new strategies for developing scoring functions in drug discovery. Neural network-based models use hand-crafted features as input, including protein–ligand interactions and ligand-dependent descriptors.^147,157,158^ Convolutional neural network (CNN)-based scoring functions adopt a different approach by using vectorized 3D grids that cover the protein–ligand binding region as input. These grids are processed through CNN layers, followed by readout layers to make predictions about the binding affinity.^159−164^ Graph neural networks (GNNs) represent molecules as graphs and can learn on the protein–ligand binding system through graph convolutions.^165−169^ However, despite the increasing popularity of DL-based scoring functions, it is worth noting that these models have not significantly outperformed traditional ML-based scoring functions on well-established benchmark data sets like CASF.^107^

Recently, Méndez-Lucio et al. proposed a geometric DL-based model named DeepDock^170^ which learns the probability density distribution of the distance between ligand atom and the protein surface. This approach showed competitive performance in docking and screening tasks when compared to those with well-established scoring functions. Building on the concept of distance likelihood, Shen et al. developed RTMScore.^171^ Unlike DeepDock, RTMScore overcomes the need for protein surface triangulation by using the probability density distribution of distances between ligand atoms and protein amino acids at the residue level. To achieve this, RTMScore represents proteins as undirect graphs at the residue level and leverages graph transformers to learn the representation of both proteins and ligands. As a result, RTMScore achieves top docking and screening power on the CASF-2016 data set, outperforming state-of-the-art ML- and DL-based scoring functions.

**Figure 3:**
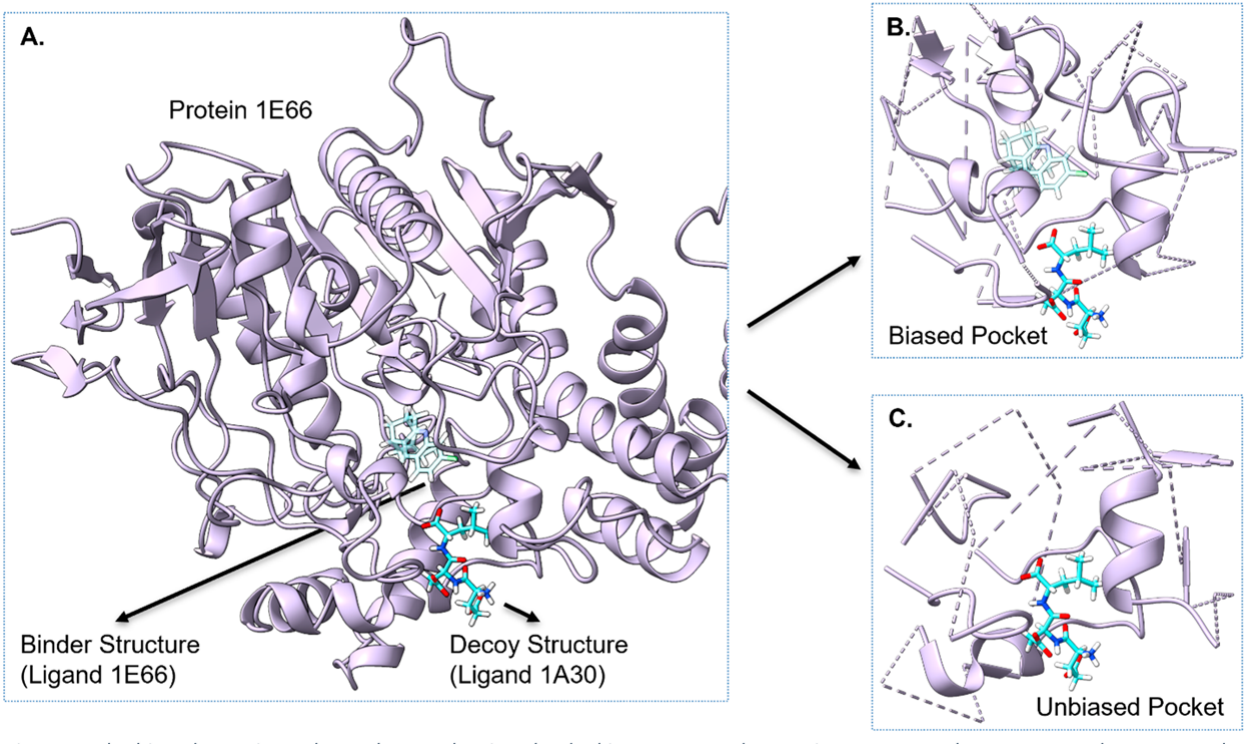
Biased protein pockets when evaluating the docking power and screening power on the CASF-2016 data set. (A) Protein and ligand structures. (B) The biased protein pocket resulting from using the binder ligand structure to cut the protein. (C) The unbiased protein pocket without using the binding structure.

However, RTMScore utilizes biased protein pockets generated from known binder structures as the input protein structure. For example, when evaluating the binding score of the ligand 1A30 on the protein 1E66 (Figure 3A), only the protein structure within 10 Å of the binder ligand 1E66 is used along with the docked ligand 1A30 structure as model input (Figure 3B). This selection method may lead to two potential problems. First, this method requires the binder structure to cut the protein, while in real world virtual screening scenarios, the protein may not have a known binder, or the binder structure may be unknown. Second, the selected pocket is biased toward the binder ligand, potentially neglecting essential interactions between the decoy ligands and the protein when the decoy structures are far from the binder ligand (Figure 3B and C).

The influence of the biased pocket selection was investigated by using docked decoy structures to cut the proteins (Figure 3C) and retesting RTMScore on the CASF-2016 data set. The results, as presented in Table 1, revealed a performance drop when the unbiased pocket was used as the model input for RTMScore models. Despite this drop in performance, the unbiased model still outperformed most of the state-of-the-art models in terms of docking and screening power. This finding highlights the importance of addressing the issue of biased pocket selection in DL-based scoring functions. Although the performance of RTMScore was affected by the unbiased pocket, the fact that it still outperformed many state-of-the-art models indicates that learning distance likelihood potential is a promising direction to improve the docking and screening performance of DL scoring functions.

**Table 1:** Docking Power and Screening Power of RTMScore on CASF-2016 Data Set When Using the Unbiased Pocket Selection Method, Compared with Other State-of-the-Art Models

	Docking Power (Success Rate)		Forward Screening Power		
Model Name	Without native poses	With native poses	EF_1%_	Top1 success rate	Reference
RTMScore1-unBiased	0.877	0.909	24.84	0.509	This work
RTMScore1-Biased	0.937	0.986	28.78	0.737	(171)
DeepDock	0.870	–	16.41	0.439	(170)
PIGNet	0.870	–	19.6	0.554	(172)
DeepBSP	0.872	0.885	–	–	(173)
OnionNet-SFCT	–	0.937	15.50	0.421	(174)
Δ_Lin___F9_XGB	–	0.867	12.61	0.404	(154)
Δ_Vina_XGB	–	0.916	13.14	0.368	(152)
Δ_Vina_RF_20_	0.849	0.891	11.73	0.421	(149)
KORP-PL	0.856	0.891	22.23	0.421	(175)
GlideScore-SP	0.846	0.877	11.44	0.368	(23)
ChemPLP@GOLD	0.832	0.860	11.91	0.351	(176)
AutoDock Vina	0.846	0.902	7.70	0.298	(22)

The very recent work by Shen et al. extends the RTMScore model to the GenScore framework.^177^ By introducing an adjustable binding affinity term during the training of the probability density distribution, GenScore successfully addresses the issue of low scoring and ranking power experienced by RTMScore, achieving balanced scoring, ranking, docking, and screening power on the CASF-2016 data set. Furthermore, the evaluation of GenScore on the LIT-PCBA data set for real-world screening power is particularly noteworthy. Their best-performing GenScore model exhibits an impressive average top 1% enhancement factor of 6.80 among all targets, demonstrating an excellent screening power.

However, it is important to acknowledge that the biased pocket problem, as mentioned earlier, is not explicitly addressed by GenScore. The reliance on pocket selection using binder ligand structures limits its applicability in real-world virtual screening protocols, especially for novel targets for which binder ligands might not be known or available. Addressing the biased pocket problem would undoubtedly enhance the utility and versatility of RTMScore, GenScore, and other DL-based scoring functions in real-world virtual screening scenarios, making them more effective in identifying potential drug candidates for novel targets with unknown binder structures. Future research in this direction may help overcome this limitation and further establish DL-based scoring functions as valuable tools in drug discovery efforts.

### 2.2 Molecular Docking in the Deep Learning Era

Traditional molecular docking methods such as AutoDock Vina^22^ and Lin_F9^139^ rely on the scoring functions and optimization algorithms that search for the global maximum of the scoring functions. The optimization algorithm rotates the rotatable bonds in the molecule, translates the molecule, and rotates the whole molecule until it finds the best conformation with the highest score predicted by the scoring function. In principle, the optimization algorithms can also be applied to DL-based scoring functions. However, in practice, the optimization algorithms often fail to converge when using DL-based scoring functions, especially for those compounds with a high number of rotatable bonds.^170^ Recently, researchers have developed DL-based docking models to predict binding poses in one shot by treating molecular docking as a regression task.^178,179^ Although these models are faster than traditional docking methods, they do not significantly outperform traditional docking methods in terms of accuracy.

In late 2022, Corso et al. introduced DiffDock,^180^ a diffusion generative model which learns the ligand pose distribution from ligand and protein structures. The model employs a diffusion process over the ligand center position, orientation, and torsional angles of each rotatable bond. By iteratively refining ligand poses through updates of translations, rotations, and torsion angles, DiffDock can sample binding poses from random initial ligand conformations.

On the PDBBind blind docking task, DiffDock achieves a 38% top-1 accuracy, significantly outperforming state-of-the-art molecular docking models.^180^ This success demonstrates the potential of DL methods in improving the ligand binding pose prediction. By combination of DiffDock with modern DL-based scoring functions, a more robust virtual screening protocol can be developed for real-world drug discovery applications.

## 3 Molecular Representation Learning with Deep Learning Methods

### 3.1 Exploring Chemical Space Efficiently with Force-Field Optimized 3D Geometry and 2D Molecular Graphs

In theoretical chemistry, the Schrödinger equation (SE) serves as the “standard model” because it represents the physics of the charged particles that make up almost all of the molecules and materials. Unfortunately, the analytical solution to SE is only available for systems with a few degrees of freedom. Numerical solutions such as high-accurate quantum mechanics (QM) calculations impose prohibitive computational cost.^181−184^ To overcome this challenge, ML approaches to predict molecular energies have been crafted to circumvent the task of solving SE of the system  by learning the mapping from the geometry to the energy  through supervised learning.^185−191^ With advances in computer infrastructure and the availability of organized chemistry data sets, DL^192^ methods are gaining popularity. DL methods utilize the atomic coordinates {***R*_*I*_**} and atom types {*Z*_*I*_} to predict molecular energetics directly, avoiding the need for hand-crafted molecular descriptors used in ML methods.^193−203^ In the past few years, many DL models^204−213^ with distinct model architectures have been developed for the prediction of molecular energetics with better performance on the QM9 benchmark data set.^214−216^ This data set contains minimized structures and molecular properties calculated using the density functional theory^217^ (DFT) method for 133,885 molecules composed of H, C, N, O, and F atoms with up to nine heavy atoms. However, despite the progress, many DL models developed on the QM9 data set still require DFT-optimized geometries as input for prediction, which leads to the original problem of computational expense not being fully addressed.

#### Force-field optimized geometries can be used to predict DFT-level molecular energetics

In 2019, Lu et al. have demonstrated the feasibility of using Merck Molecular Force Field (MMFF)^183^ optimized geometry for gas phase DFT-level electronic energy prediction.^209^ They first train a model called DTNN_7ib on the QM9 data set using DFT-optimized geometries as input and then retrain the readout layer of the model on their prepared MMFF-optimized geometries. The trained DTNN_7ib achieves a mean-absolute-error (MAE) of 0.79 kcal/mol on DFT-level electronic energy by using MMFF-optimized geometry.

In 2021, Lu et al. extended the effectiveness of MMFF-optimized geometry beyond the QM9 benchmark data set by constructing the Frag20 data set.^63^ These data consisted of DFT-calculated properties for over half a million molecules, with both MMFF and DFT-optimized geometries, and consisted of elements H, B, C, O, N, F, P, S, Cl, and Br, with no more than 20 heavy atoms. They train a model named sPhysNet on the Frag20 data set and achieve better than or close to chemical accuracy (1 kcal/mol) on multiple test sets, including two external test sets based on experimental crystal structures. The work indicated the potential of using MMFF-optimized geometries as a more computationally efficient alternative to explore chemical space^66^ compared to solely relying on DFT-optimized geometries.

#### MMFF geometry also proves useful for predicting experimental properties

**Figure 4:**
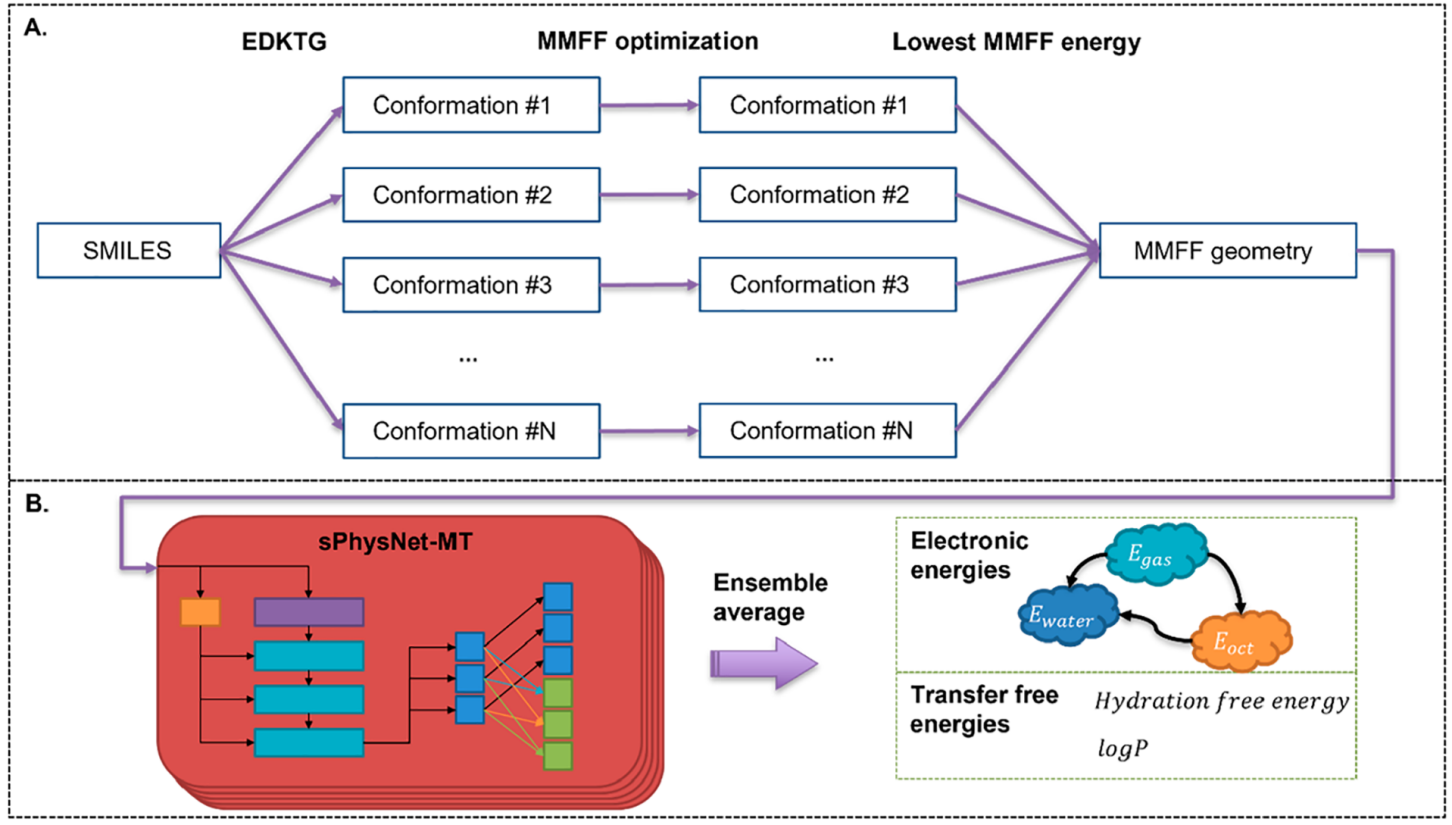
Overall workflow using sPhysNet-MT-ens5 for energetics prediction. (A) MMFF geometry generation protocol. (B) The prediction of electronic energies and transfer free energies with sPhysNet-MT-ens5.

In 2022, Xia et al.^51^ developed a DL model named sPhysNet-MT-ens5 for predicting experimental hydration free energy and octanol/water partition coefficient (logP) using MMFF optimized geometry. Figure 4 shows the overall workflow using the model for prediction. For each compound, up to 300 conformations are generated using EDKTG^218^ implemented in RDKit^219^ and then optimized with MMFF. The conformation with the lowest MMFF energy is then selected as the input geometry for the prediction of experimental hydration free energy and logP, as well as other properties of the compound. An ensemble of five independently trained sPhysNet-MT models is used for prediction, and the average of the five predictions is used as the ensemble prediction. The model achieves a root-mean-square error (RMSE) of 0.620 kcal/mol on the FreeSolv^220^ data set for experimental hydration free energy prediction, as well as an RMSE of 0.393 on the PHYSPROP^18,221−223^ data set for experimental logP prediction.

#### Spatial information computed from MMFF geometry enhances 2D molecular representations

Unlike models that use 3D molecular geometry as input, many DL models use 2D molecular graphs to predict various chemical properties.^224−231^ While they have shown success in these tasks, their limitations in representing molecules with 2D graphs have been widely discussed.^232−236^ This is because models based solely on 2D connection graphs may not distinguish between molecules with 3D spatial structures. For example, message passing GNNs cannot distinguish decaprismane^237^ C_20_H_20_ from dodecahedrane^238^ C_20_H_20_ purely based on their 2D graphs.^235^

To address this limitation, models that incorporate geometry information can achieve better performance.^52,239^ In 2022, Zhang et al. developed A3D-PNAConv^52^ which uses both 2D and 3D information on molecules. They demonstrate that incorporating 3D atomic features obtained from MMFF-optimized geometries enhances the learning power of GNN models in predicting calculated solvation free energies. The 3D atomic features are calculated by atom-centered symmetry functions (ACSFs),^200^ which are sets of many-body interaction functions encoding atomic environments in 3D structures. The GNNs use the 3D atomic features as the initial atomic embedding and then perform several iterations of message passing on the 2D molecular graphs. In comparison, they have also trained GNNs without 3D atomic features. The GNNs with 3D atomic features as initialization outperform their 2D counterpart for predicting calculated water solvation free energy on their Frag20-Aqsol-100k data set. This indicates that using 3D atomic features obtained from MMFF geometries can enhance the learning capability of GNN-based models.

### 3.2 Enhancing Downstream Tasks through Transfer Learning with Pretrained Molecular Modeling Data

In the early stages of the drug discovery process, data scarcity is the most common issue for DL models in the prediction of experimental physicochemical properties. DL models heavily rely on the size and quality of the training data, and they tend to overfit on small data sets, leading to poor generalizability and performance.^240^ In contrast, molecular modeling data can be obtained in larger quantities within a reasonable time frame, thanks to advancements in high-performance computing. As shown in Table 2, publicly available data sets generated using molecular modeling methods are significantly larger than experimental data sets in terms of size. To address the scarcity of experimental values, calculated data sets should be fully utilized when training DL models on experimental data.

**Table 2:** Overview of Common Experimental and Calculated Data Sets for Energetic-Related Properties

Type	Name	Data set size	#Heavy Atoms	Properties	Reference
Calculated	QM9	133,885	1–9	Electronic energy, HOMO, LUMO, etc.	(215)
	ANI-1	24,687,809	1–8	Electronic energy	(241)
	Frag20	566,296	1–20	Electronic energy	(63)
	Frag20-solv-678k	678,916	1–20	Electronic energies (gas water and octanol)	(51)
Experimental	FreeSolv	643	1–24	Hydration Free Energy	(220)
	PHYSPROP-logP	14,174	3–85	Water/octanol partition coefficient	(221,223)
	MNSOL	3,037	0–28	Solvation free energies and transfer free energies in 92 solvents	(242)

#### Pretraining on DFT-calculated energetics aids in improving the prediction of downstream tasks on related experimental properties

The fine-tuning strategy is commonly employed in training DL models. First, a model is pretrained on a source task, and then, its trained weights are transferred as the initialization for a related target task. This fine-tuning approach often yields better performance compared to random initialization.^243^ For instance, Zhang et al. developed a data set named Frag20-Aqsol-100k which contains 100k diverse molecules sampled from Frag20 and CSD20 data sets, along with their calculated hydration free energy using their SMD_B3LYP protocol.^52^ They pretrained DL models on the Frag20-Aqsol-100k data set and fine-tuned the models on the FreeSolv data set, which contains 640 compounds with experimentally measured hydration free energy. Their fine-tuned model achieved state-of-the-art performance and can even achieve chemical accuracy with a very small training data size of just 100 molecules.^52^

**Figure 5:**
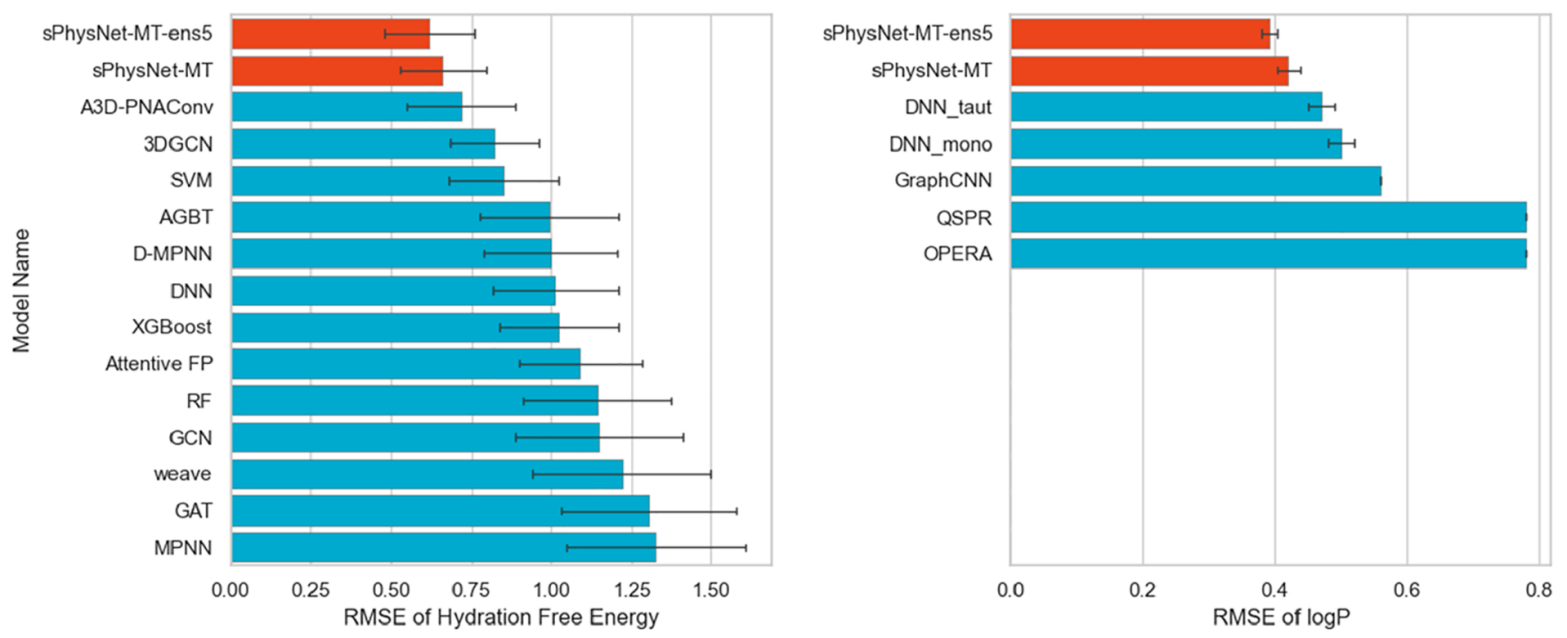
Model performance comparison between sPhysNet-MT and other state-of-the-art models on the experimental hydration free energy and logP prediction. Results are adapted from the sPhysNet-MT manuscript.^51^

Xia et al.^51^ extended the work and develop Frag20-solv-678k which contains 678,916 conformations and their calculated energetics in gas, water, and octanol phases. They pretrained a DL model on all three electronic energetics and the transfer free energies between the three phases, and fine-tuned the model on a combined data set composed of molecules from FreeSolv with experimental hydration free energy and PHYSPROP with experimental logP. The fine-tuned model predicts experimental hydration free energy with an RMSE of 0.620 kcal/mol on the FreeSolv data set, as well as experimental logP with an RMSE of 0.393 on the PHYSPROP data set, achieving state-of-the-art performance on both tasks simultaneously (Figure 5).

### 3.3 Improving Molecular Representations through Mutual Learning with Multitask Training

There are a wide range of ML and DL models that have been developed for a variety of chemical tasks. Although some chemical tasks are related, they are treated by different models with distinct model architectures. For example, the models developed for the prediction of high-level QM calculated molecular energetics^204−212^ and the models for predicting of experimental transfer free energies^52,225,228,229,239,244,245^ are often developed separately. While separate models may be more effective for accurate predictions for weakly related tasks,^210,211^ multitask models can be advantageous when tasks are well-related.^246,247^

#### Multitask training on related tasks leads to more robust molecular representation learning

Motivated by the fact that many molecular modeling methods use the direct relationship between absolute energies and transfer energies to predict experimental properties with satisfactory results,^248−251^ Xia et al. developed a multitask DL model named sPhysNet-MT.^51^ This model simultaneously predicts electronic energies of molecules in gas, water, and octanol as well as the transfer free energies between these phases. On their calculated data set, which includes molecules with energetics computed at the DFT-level, the sPhysNet-MT model achieves chemical accuracy on all tasks. Additionally, on the experimental data set containing molecules with experimental hydration free energy and logP, the model achieves state-of-the-art performance on both tasks simultaneously (Figure 5).

Similarly, Lu et al. developed the T5Chem model for multitask reaction prediction, which encompasses forward reaction prediction, reactant prediction, reagent prediction, reaction yield prediction, and reaction type classification.^252^ On the test set of their reaction prediction data set called USPTO_500_MT, T5Chem achieves top-1 accuracy of 97.5%, 72.9%, and 24.9% for forward prediction, retrosynthesis, and reagents prediction, respectively. The model also achieves an accuracy of 99.4% in reaction type classification, *R*^2^ of 0.22, and MAE of 17.8 in reaction yield prediction, showing competitive performance compared to models trained solely on individual tasks.

## 4 Case Study: 1M17

**Figure 6:**
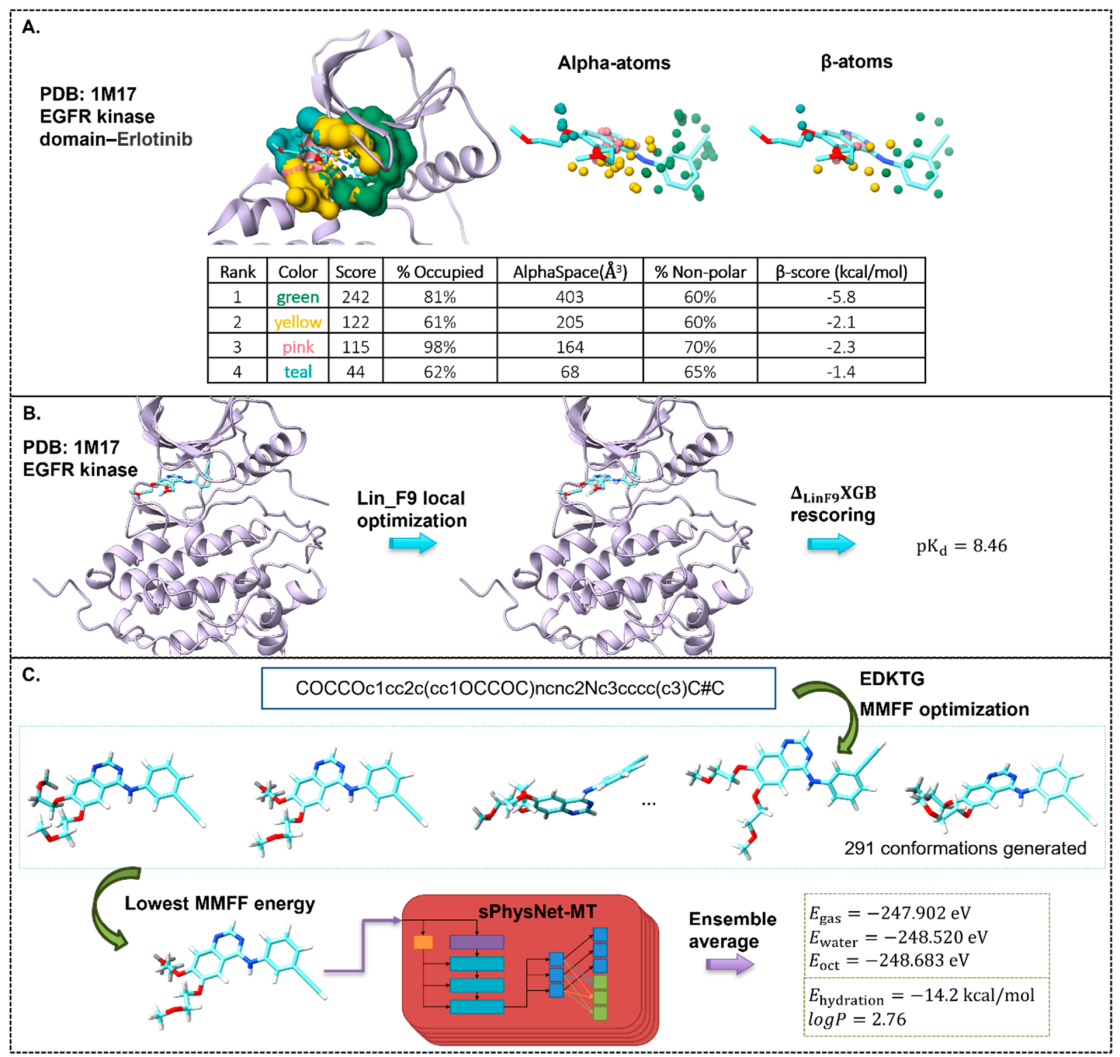
Case study of PDB 1M17 with models mentioned in the paper. (A) AlphaSpace and AlphaSpace 2.0 binding pocket analysis. (B) Binding affinity prediction was done using Lin_F9 and Δ_*LinF*9_*XGB*. (C) Prediction of physicochemical properties of the ligand molecule using sPhysNet-MT.

To illustrate the computational tools described in this paper, we show a case study using the crystal structure of the FDA approved inhibitor Erlotinib bound to the kinase domain of the epidermal growth factor receptor (EGFR) from PDB 1M17 (Figure 6).

We first use AlphaSpace to analyze the binding of Erlotinib to EGFR kinase. The crystal receptor and crystal ligand are the inputs for AlphaSpace and AlphaSpace2.0 analysis. To calculate the AlphaSpace 2.0 β-score, hydrogens and partial charges are added to the ligand and protein input files. The AlphaSpace output characterizes the pockets that are in contact with the ligand, provides a binding score, and suggests undertargeted pockets (Figure 6A). The alpha-atoms and β-atoms are colored by the pockets which they belong to.

We then apply Δ_Lin_F9_XGB for the prediction of the binding affinity. Similarly to the preparation of the AlphaSpace 2.0 input files, we add hydrogens and generate the partial charges to the crystal ligand and crystal protein input files. We apply Lin_F9 local optimization on the ligand binding orientation and get the Lin_F9 binding score. The Vina, solvent accessible surface area, water, metal, and ligand descriptors are calculated for the optimized geometry. These are input to an ensemble of 10 XGB models, the average output of which is used as correction of the Lin_F9 binding affinity. The sum of the Lin_F9 and XGB correction is the final Δ_Lin_F9_XGB score or the predicted binding affinity. The predicted binding affinity of Erlotnib to EGFR kinase from the structure 1M17 (p*K*_d_^pred^ = 8.46, *K*_d_^pred^ = 3.5 nM, Figure 6B) closely matches the experimental binding affinity found in BindingDB (with an average of p*K*_d_^exp^ and p*K*_i_^exp^ = 8.9 ± 0.9).^253^ It is worth noting that BindingDB contains 45 entries for *K*_i_ (ranging from 0.1 to 136 nM) and *K*_d_ (ranging from 0.35 to 190 nM), indicating a wide range of experimentally measured binding affinities. Our predicted *K*_d_ value of 3.5 nM falls comfortably within this spectrum of binding affinities.

Finally, we use sPhysNet-MT to predict the physicochemical properties of the ligand molecule Erlotinib. Starting from the SMILES of Erlotinib, we first generate up to 300 conformations using EDKTG and optimize them with MMFF. 291 conformations are successfully generated, and the one with the lowest MMFF energy is selected as the input geometry for sPhysNet-MT. The average output of an ensemble of 5 independently trained sPhysNet-MT models is used as the final prediction. The model predicts the logP of the molecule to be 2.76 which is close to the experimental measured value queried from the DrugBank database, 2.7 (Figure 6C).^254^ The detailed scripts to carry out these calculations and analysis can be found at: https://github.com/SongXia-NYU/drug_discovery_perspective.

## 5 Concluding Remarks and Perspective

The success of developing computational tools for drug design requires the integration of both molecular modeling and ML techniques. Models for small molecules, protein surfaces, and protein–ligand interactions are all required during different stages of the drug design process, and there is still room for development and research in these methods.

The AlphaSpace methods can be more robust and applicable. Further improvements can account for the role of protein conformational stability in pocket ligand-ability. This can make pocket comparisons more robust, especially when large conformational changes or comparisons between *apo* and *holo* structures are involved. When *apo* structures result in lower ligand-ability estimates, it limits confidence in using *apo* structures for downstream structure-based design. Quantified comparisons between different proteins and between orthosteric and allosteric pockets can also provide insights into ligand selectivity and off-target effects. Future versions can address these limitations by leveraging deep learning approaches to learn the representation of these relationships and improve ligand-ability estimates. Smith et al. suggest that there are opportunities to apply advanced deep learning image vision techniques to this research question.^92^ AlphaSpace2 and future versions can be tested on recently developed benchmarks for protein–ligand binding site prediction such as SHREC,^83^ HOLO4K, COACH420,^78^ and for binding site comparisons such as ProSPECCTs.^70,255^ Additionally, a Web server can be developed to give medicinal chemists a more user-friendly experience.

Despite the improved performance of ML- and DL-based molecular docking and scoring methods on the CASF-2016 data set, these methods tend to have a high false positive rate and lack generalizability on real-world VS data sets. More consideration should be given to the curation of the training data sets and to introducing regularization techniques to current VS protocol. Computationally generated decoy poses and training data sets should be generated carefully to prevent the model from having noncausal bias, an issue where the model learns specific data patterns instead of meaningful ligand–protein interactions.^256,257^ Training data sets tend to have many positive labels that lack diversity and have few or biased negative labels.^83,88,258,259^ Non- and poor-binders are under-reported, and computationally selected nonbinders should be verified experimentally.^260,261^ There is a lack of quality and standardized data for some targets. Methods such as data set debiasing^262^ and introducing specific models to classify and rank actives from inactives^171,263−265^ are promising regularization techniques to tackle this problem. Furthermore, it is necessary to test models in fair benchmarking data sets akin to real-world conditions.^155,266^

The size and quality of the training data are also important considerations for the prediction of molecular properties with DL models. More diverse data sets are required to further explore the chemical space. The Frag20 data preparation protocol^63^ can be used to carefully select diverse molecules, and modern high performance computing platforms can with the help generate labels for DFT calculations for calculated data sets. Collaboration with experimental groups is needed to enlarge and standardize experimental training sets. More careful pretraining protocols and model architecture designs should be developed to fully take advantage of the existing experimental data. Another disadvantage of sPhysNet-MT as well as many other DL-based models is that they can run into problems when considering charged molecules, because they do not explicitly express molecular charge in the model. For example, when predicting p*K*_a_, the molecule may be ionized and the model may have problems learning the molecular representation without knowing the total charge of the system.

Additionally, structure-based drug design methods are limited by the exclusion of protein dynamics in most current protein–ligand representations. Protein flexibility can range from slight perturbation of the ligand binding pockets to reconstitution of the binding site to large-scale conformational changes. In the case of binding site prediction, the introduction of the protein dynamics to the model can lead to the detection of cryptic pockets that are not seen in the static crystal structures and to better predictors of binding site ligandability.^82^ Cryptic pockets can be teased out using standard MD simulations or specialized MD simulations where the solvent molecules are altered to act as probes to tease out these pockets.^80^ Researchers are also using ML models that have learned the aspects of protein flexibility that lead to cryptic pockets from MD simulations to predict cryptic sites.^267,268^

Researchers have addressed this limitation of protein flexibility within the context of docking and binding affinity prediction as well.^269^ Some methods introduce side chain flexibility to the receptor binding pocket,^270^ others include MD simulations to probe the stability of the predicted binding pose,^271^ while some DL methods learn the features that drive protein–ligand interactions in MD simulations to predict binding affinity.^272^ The most common method is ensemble docking, which performs docking on a diverse selection of static receptor structures.^273,274^ Although rigorous in concept, ensemble docking can actually lead to a decrease in performance when using too many structures and thus researchers propose additional ML methods to select the best subset of structures.^275−277^ The use of technology, like cryo-EM that can determine diverse receptor structures, enhanced sampling MD simulations that speed conformational sampling, and DL protein structure prediction methods that can predict previously undetermined protein structures and conformations will drive the research in understanding the role of protein conformation in ligand binding. The decision to introduce protein flexibility to structure-based drug design comes with additional computational cost, a factor that must be aligned with the scope and goals of every screening campaign and currently does not guarantee improved performance.

## References

[ref1] Brown D. G.; Wobst H. J. A decade of FDA-approved drugs (2010–2019): trends and future directions. Journal of medicinal chemistry 2021, 64 (5), 2312–2338. 10.1021/acs.jmedchem.0c01516. 33617254

[ref2] Mullard A. 2020 FDA drug approvals. Nat. Rev. Drug Discovery 2021, 20 (2), 85–91. 10.1038/d41573-021-00002-0. 33402709

[ref3] Wouters O. J.; McKee M.; Luyten J. Research and development costs of new drugs—reply. JAMA 2020, 324 (5), 518–518. 10.1001/jama.2020.8651. 32749488

[ref4] Shaker B.; Ahmad S.; Lee J.; Jung C.; Na D. In silico methods and tools for drug discovery. Computers in Biology and Medicine 2021, 137, 104851 10.1016/j.compbiomed.2021.104851. 34520990

[ref5] Katsila T.; Spyroulias G. A.; Patrinos G. P.; Matsoukas M.-T. Computational approaches in target identification and drug discovery. Computational and Structural Biotechnology Journal 2016, 14, 177–184. 10.1016/j.csbj.2016.04.004. 27293534 PMC4887558

[ref6] Ekins S.; Puhl A. C.; Zorn K. M.; Lane T. R.; Russo D. P.; Klein J. J.; Hickey A. J.; Clark A. M. Exploiting machine learning for end-to-end drug discovery and development. Nat. Mater. 2019, 18 (5), 435–441. 10.1038/s41563-019-0338-z. 31000803 PMC6594828

[ref7] Zhavoronkov A. Artificial Intelligence for Drug Discovery, Biomarker Development, and Generation of Novel Chemistry. Mol. Pharmaceutics 2018, 15 (10), 4311–4313. 10.1021/acs.molpharmaceut.8b00930. 30269508

[ref8] Gupta R.; Srivastava D.; Sahu M.; Tiwari S.; Ambasta R. K.; Kumar P. Artificial intelligence to deep learning: machine intelligence approach for drug discovery. Molecular Diversity 2021, 25 (3), 1315–1360. 10.1007/s11030-021-10217-3. 33844136 PMC8040371

[ref9] Vijayan R. S. K.; Kihlberg J.; Cross J. B.; Poongavanam V. Enhancing preclinical drug discovery with artificial intelligence. Drug Discovery Today 2022, 27 (4), 967–984. 10.1016/j.drudis.2021.11.023. 34838731

[ref10] Patel L.; Shukla T.; Huang X.; Ussery D. W.; Wang S. Machine learning methods in drug discovery. Molecules 2020, 25 (22), 5277 10.3390/molecules25225277. 33198233 PMC7696134

[ref11] Chandrasekaran B.; Abed S. N.; Al-Attraqchi O.; Kuche K.; Tekade R. K. Chapter 21 - Computer-Aided Prediction of Pharmacokinetic (ADMET) Properties. In Dosage Form Design Parameters; Tekade R. K., Ed.; Academic Press: 2018; pp 731–755.

[ref12] Wagner J. A.; Dahlem A. M.; Hudson L. D.; Terry S. F.; Altman R. B.; Gilliland C. T.; DeFeo C.; Austin C. P. Application of a dynamic map for learning, communicating, navigating, and improving therapeutic development. Clinical and Translational Science 2018, 11 (2), 166–174. 10.1111/cts.12531. 29271559 PMC5866991

[ref13] Hughes J. P.; Rees S.; Kalindjian S. B.; Philpott K. L. Principles of early drug discovery. British journal of pharmacology 2011, 162 (6), 1239–1249. 10.1111/j.1476-5381.2010.01127.x. 21091654 PMC3058157

[ref14] Acharya C.; Coop A.; Polli J. E.; MacKerell A. D. Recent advances in ligand-based drug design: relevance and utility of the conformationally sampled pharmacophore approach. Current computer-aided drug design 2011, 7 (1), 10–22. 10.2174/157340911793743547. 20807187 PMC2975775

[ref15] Bender A.; Jenkins J. L.; Scheiber J.; Sukuru S. C. K.; Glick M.; Davies J. W. How similar are similarity searching methods? A principal component analysis of molecular descriptor space. J. Chem. Inf. Model. 2009, 49 (1), 108–119. 10.1021/ci800249s. 19123924

[ref16] Wolber G.; Langer T. LigandScout: 3-D pharmacophores derived from protein-bound ligands and their use as virtual screening filters. J. Chem. Inf. Model. 2005, 45 (1), 160–169. 10.1021/ci049885e. 15667141

[ref17] Chen J.; Lai L. Pocket v. 2: further developments on receptor-based pharmacophore modeling. J. Chem. Inf. Model. 2006, 46 (6), 2684–2691. 10.1021/ci600246s. 17125208

[ref18] Leo A.; Hoekman D. Exploring QSAR; American Chemical Society: 1995.

[ref19] Verma J.; Khedkar V. M.; Coutinho E. C. 3D-QSAR in drug design-a review. Current topics in medicinal chemistry 2010, 10 (1), 95–115. 10.2174/156802610790232260. 19929826

[ref20] Yuriev E.; Agostino M.; Ramsland P. A. Challenges and advances in computational docking: 2009 in review. Journal of Molecular Recognition 2011, 24 (2), 149–164. 10.1002/jmr.1077. 21360606

[ref21] Verdonk M. L.; Cole J. C.; Hartshorn M. J.; Murray C. W.; Taylor R. D. Improved protein-ligand docking using GOLD. Proteins: Struct., Funct., Bioinf. 2003, 52 (4), 609–623. 10.1002/prot.10465. 12910460

[ref22] Trott O.; Olson A. J. AutoDock Vina: improving the speed and accuracy of docking with a new scoring function, efficient optimization, and multithreading. Journal of computational chemistry 2010, 31 (2), 455–461. 10.1002/jcc.21334. 19499576 PMC3041641

[ref23] Friesner R. A.; Banks J. L.; Murphy R. B.; Halgren T. A.; Klicic J. J.; Mainz D. T.; Repasky M. P.; Knoll E. H.; Shelley M.; Perry J. K.; et al. Glide: A New Approach for Rapid, Accurate Docking and Scoring. 1. Method and Assessment of Docking Accuracy. J. Med. Chem. 2004, 47 (7), 1739–1749. 10.1021/jm0306430. 15027865

[ref24] Jain A. N. Surflex: fully automatic flexible molecular docking using a molecular similarity-based search engine. Journal of medicinal chemistry 2003, 46 (4), 499–511. 10.1021/jm020406h. 12570372

[ref25] Jain A. N. Surflex-Dock 2.1: Robust performance from ligand energetic modeling, ring flexibility, and knowledge-based search. Journal of Computer-Aided Molecular Design 2007, 21 (5), 281–306. 10.1007/s10822-007-9114-2. 17387436

[ref26] Miteva M. A.; Lee W. H.; Montes M. O.; Villoutreix B. O. Fast structure-based virtual ligand screening combining FRED, DOCK, and Surflex. Journal of medicinal chemistry 2005, 48 (19), 6012–6022. 10.1021/jm050262h. 16162004

[ref27] Hansson T.; Oostenbrink C.; van Gunsteren W. Molecular dynamics simulations. Curr. Opin. Struct. Biol. 2002, 12 (2), 190–196. 10.1016/S0959-440X(02)00308-1. 11959496

[ref28] Kaya S.; Tüzün B.; Kaya C.; Obot I. B. Determination of corrosion inhibition effects of amino acids: quantum chemical and molecular dynamic simulation study. Journal of the Taiwan Institute of Chemical Engineers 2016, 58, 528–535. 10.1016/j.jtice.2015.06.009.

[ref29] Pronk S.; Páll S.; Schulz R.; Larsson P.; Bjelkmar P.; Apostolov R.; Shirts M. R.; Smith J. C.; Kasson P. M.; Van Der Spoel D. GROMACS 4.5: a high-throughput and highly parallel open source molecular simulation toolkit. Bioinformatics 2013, 29 (7), 845–854. 10.1093/bioinformatics/btt055. 23407358 PMC3605599

[ref30] Nelson M. T.; Humphrey W.; Gursoy A.; Dalke A.; Kalé L. V.; Skeel R. D.; Schulten K. NAMD: a parallel, object-oriented molecular dynamics program. International Journal of Supercomputer Applications and High Performance Computing 1996, 10 (4), 251–268. 10.1177/109434209601000401.

[ref31] Pearlman D. A.; Case D. A.; Caldwell J. W.; Ross W. S.; Cheatham T. E. III; DeBolt S.; Ferguson D.; Seibel G.; Kollman P. AMBER, a package of computer programs for applying molecular mechanics, normal mode analysis, molecular dynamics and free energy calculations to simulate the structural and energetic properties of molecules. Comput. Phys. Commun. 1995, 91 (1–3), 1–41. 10.1016/0010-4655(95)00041-D.

[ref32] Jo S.; Kim T.; Iyer V. G.; Im W. CHARMM-GUI: a web-based graphical user interface for CHARMM. Journal of computational chemistry 2008, 29 (11), 1859–1865. 10.1002/jcc.20945. 18351591

[ref33] Katigbak J.; Li H.; Rooklin D.; Zhang Y. AlphaSpace 2.0: Representing Concave Biomolecular Surfaces Using β-Clusters. J. Chem. Inf. Model. 2020, 60 (3), 1494–1508. 10.1021/acs.jcim.9b00652. 31995373 PMC7093224

[ref34] Rooklin D.; Wang C.; Katigbak J.; Arora P. S.; Zhang Y. AlphaSpace: Fragment-Centric Topographical Mapping To Target Protein-Protein Interaction Interfaces. J. Chem. Inf. Model. 2015, 55 (8), 1585–1599. 10.1021/acs.jcim.5b00103. 26225450 PMC4550072

[ref35] Le Guilloux V.; Schmidtke P.; Tuffery P. Fpocket: An open source platform for ligand pocket detection. BMC Bioinformatics 2009, 10 (1), 168 10.1186/1471-2105-10-168. 19486540 PMC2700099

[ref36] Tian W.; Chen C.; Lei X.; Zhao J.; Liang J. CASTp 3.0: computed atlas of surface topography of proteins. Nucleic acids research 2018, 46 (W1), W363–W367. 10.1093/nar/gky473. 29860391 PMC6031066

[ref37] Halgren T. A. Identifying and characterizing binding sites and assessing druggability. J. Chem. Inf Model 2009, 49 (2), 377–389. 10.1021/ci800324m. 19434839

[ref38] Wass M. N.; Kelley L. A.; Sternberg M. J. 3DLigandSite: predicting ligand-binding sites using similar structures. Nucleic acids research 2010, 38 (suppl_2), W469–W473. 10.1093/nar/gkq406. PMC2896164 20513649

[ref39] Kalidas Y.; Chandra N. PocketDepth: a new depth based algorithm for identification of ligand binding sites in proteins. J. Struct. Biol. 2008, 161 (1), 31–42. 10.1016/j.jsb.2007.09.005. 17949996

[ref40] Eswar N.; Eramian D.; Webb B.; Shen M.-Y.; Sali A. Protein structure modeling with MODELLER. Structural proteomics: high-throughput methods 2008, 426, 145–159. 10.1007/978-1-60327-058-8_8. 18542861

[ref41] Waterhouse A.; Bertoni M.; Bienert S.; Studer G.; Tauriello G.; Gumienny R.; Heer F. T.; de Beer T. A. P.; Rempfer C.; Bordoli L. SWISS-MODEL: homology modelling of protein structures and complexes. Nucleic acids research 2018, 46 (W1), W296–W303. 10.1093/nar/gky427. 29788355 PMC6030848

[ref42] Pieper U.; Eswar N.; Davis F. P.; Braberg H.; Madhusudhan M. S.; Rossi A.; Marti-Renom M.; Karchin R.; Webb B. M.; Eramian D. MODBASE: a database of annotated comparative protein structure models and associated resources. Nucleic acids research 2006, 34 (90001), D291–D295. 10.1093/nar/gkj059. 16381869 PMC1347422

[ref43] Kelley L. A.; Mezulis S.; Yates C. M.; Wass M. N.; Sternberg M. J. The Phyre2 web portal for protein modeling, prediction and analysis. Nature protocols 2015, 10 (6), 845–858. 10.1038/nprot.2015.053. 25950237 PMC5298202

[ref44] Hardin C.; Pogorelov T. V.; Luthey-Schulten Z. Ab initio protein structure prediction. Curr. Opin. Struct. Biol. 2002, 12 (2), 176–181. 10.1016/S0959-440X(02)00306-8. 11959494

[ref45] Yang J.; Zhang Y. I-TASSER server: new development for protein structure and function predictions. Nucleic acids research 2015, 43 (W1), W174–W181. 10.1093/nar/gkv342. 25883148 PMC4489253

[ref46] Kim D. E.; Chivian D.; Baker D. Protein structure prediction and analysis using the Robetta server. Nucleic acids research 2004, 32 (suppl_2), W526–W531. 10.1093/nar/gkh468. 15215442 PMC441606

[ref47] Xu D.; Zhang J.; Roy A.; Zhang Y. Automated protein structure modeling in CASP9 by I-TASSER pipeline combined with QUARK-based ab initio folding and FG-MD-based structure refinement. Proteins: Struct., Funct., Bioinf. 2011, 79 (S10), 147–160. 10.1002/prot.23111. PMC3228277 22069036

[ref48] Kennedy T. Managing the drug discovery/development interface. Drug discovery today 1997, 2 (10), 436–444. 10.1016/S1359-6446(97)01099-4.

[ref49] Tetko I. V.; Tanchuk V. Y. Application of associative neural networks for prediction of lipophilicity in ALOGPS 2.1 program. Journal of chemical information and computer sciences 2002, 42 (5), 1136–1145. 10.1021/ci025515j. 12377001

[ref50] Nunes A. M. V.; de Andrade F. d. C. P.; Filgueiras L. A.; de Carvalho Maia O. A.; Cunha R. L.; Rodezno S. V.; Maia Filho A. L. M.; de Amorim Carvalho F. A.; Braz D. C.; Mendes A. N. preADMET analysis and clinical aspects of dogs treated with the Organotellurium compound RF07: A possible control for canine visceral leishmaniasis?. Environmental Toxicology and Pharmacology 2020, 80, 103470 10.1016/j.etap.2020.103470. 32814174

[ref51] Xia S.; Zhang D.; Zhang Y. Multitask Deep Ensemble Prediction of Molecular Energetics in Solution: From Quantum Mechanics to Experimental Properties. J. Chem. Theory Comput. 2023, 19 (2), 659–668. 10.1021/acs.jctc.2c01024. PMC10323048 36607141

[ref52] Zhang D.; Xia S.; Zhang Y. Accurate Prediction of Aqueous Free Solvation Energies Using 3D Atomic Feature-Based Graph Neural Network with Transfer Learning. J. Chem. Inf. Model. 2022, 62 (8), 1840–1848. 10.1021/acs.jcim.2c00260. 35422122 PMC9038704

[ref53] Daina A.; Michielin O.; Zoete V. SwissADME: a free web tool to evaluate pharmacokinetics, drug-likeness and medicinal chemistry friendliness of small molecules. Sci. Rep 2017, 7 (1), 42717 10.1038/srep42717. 28256516 PMC5335600

[ref54] Dhanda S. K.; Singla D.; Mondal A. K.; Raghava G. P. DrugMint: a webserver for predicting and designing of drug-like molecules. Biology Direct 2013, 8 (1), 1–12. 10.1186/1745-6150-8-28. 24188205 PMC3826839

[ref55] Shaker B.; Yu M.-S.; Song J. S.; Ahn S.; Ryu J. Y.; Oh K.-S.; Na D. LightBBB: computational prediction model of blood-brain-barrier penetration based on LightGBM. Bioinformatics 2021, 37 (8), 1135–1139. 10.1093/bioinformatics/btaa918. 33112379

[ref56] Lee H.-M.; Yu M.-S.; Kazmi S. R.; Oh S. Y.; Rhee K.-H.; Bae M.-A.; Lee B. H.; Shin D.-S.; Oh K.-S.; Ceong H. Computational determination of hERG-related cardiotoxicity of drug candidates. BMC bioinformatics 2019, 20, 67–73. 10.1186/s12859-019-2814-5. 31138104 PMC6538553

[ref57] Gupta S.; Kapoor P.; Chaudhary K.; Gautam A.; Kumar R.; Consortium O. S. D. D.; Raghava G. P. In silico approach for predicting toxicity of peptides and proteins. PloS one 2013, 8 (9), e73957 10.1371/journal.pone.0073957. 24058508 PMC3772798

[ref58] Banerjee P.; Eckert A. O.; Schrey A. K.; Preissner R. ProTox-II: a webserver for the prediction of toxicity of chemicals. Nucleic acids research 2018, 46 (W1), W257–W263. 10.1093/nar/gky318. 29718510 PMC6031011

[ref59] Mishra N. K.; Singla D.; Agarwal S.; Raghava G. P. ToxiPred: a server for prediction of aqueous toxicity of small chemical molecules in T. Pyriformis. Journal of Translational Toxicology 2014, 1 (1), 21–27. 10.1166/jtt.2014.1005.

[ref60] Dong J.; Wang N.-N.; Yao Z.-J.; Zhang L.; Cheng Y.; Ouyang D.; Lu A.-P.; Cao D.-S. ADMETlab: a platform for systematic ADMET evaluation based on a comprehensively collected ADMET database. Journal of cheminformatics 2018, 10, 1–11. 10.1186/s13321-018-0283-x. 29943074 PMC6020094

[ref61] Skinnider M. A.; Stacey R. G.; Wishart D. S.; Foster L. J. Chemical language models enable navigation in sparsely populated chemical space. Nature Machine Intelligence 2021, 3 (9), 759–770. 10.1038/s42256-021-00368-1.

[ref62] Mahmood O.; Mansimov E.; Bonneau R.; Cho K. Masked graph modeling for molecule generation. Nat. Commun. 2021, 12 (1), 3156 10.1038/s41467-021-23415-2. 34039973 PMC8155025

[ref63] Lu J.; Xia S.; Lu J.; Zhang Y. Dataset Construction to Explore Chemical Space with 3D Geometry and Deep Learning. J. Chem. Inf. Model. 2021, 61 (3), 1095–1104. 10.1021/acs.jcim.1c00007. 33683885 PMC8012661

[ref64] Dou B.; Zhu Z.; Merkurjev E.; Ke L.; Chen L.; Jiang J.; Zhu Y.; Liu J.; Zhang B.; Wei G.-W. Machine Learning Methods for Small Data Challenges in Molecular Science. Chem. Rev. 2023, 123 (13), 8736–8780. 10.1021/acs.chemrev.3c00189. 37384816 PMC10999174

[ref65] Sadybekov A. V.; Katritch V. Computational approaches streamlining drug discovery. Nature 2023, 616 (7958), 673–685. 10.1038/s41586-023-05905-z. 37100941

[ref66] Reymond J.-L. The Chemical Space Project. Acc. Chem. Res. 2015, 48 (3), 722–730. 10.1021/ar500432k. 25687211

[ref67] Nisius B.; Sha F.; Gohlke H. Structure-based computational analysis of protein binding sites for function and druggability prediction. J. Biotechnol. 2012, 159 (3), 123–134. 10.1016/j.jbiotec.2011.12.005. 22197384

[ref68] Macari G.; Toti D.; Polticelli F. Computational methods and tools for binding site recognition between proteins and small molecules: from classical geometrical approaches to modern machine learning strategies. Journal of Computer-Aided Molecular Design 2019, 33 (10), 887–903. 10.1007/s10822-019-00235-7. 31628659

[ref69] Rehman A. U.; Lu S.; Khan A. A.; Khurshid B.; Rasheed S.; Wadood A.; Zhang J. Hidden allosteric sites and De-Novo drug design. Expert Opinion on Drug Discovery 2022, 17 (3), 283–295. 10.1080/17460441.2022.2017876. 34933653

[ref70] Ehrt C.; Brinkjost T.; Koch O. A benchmark driven guide to binding site comparison: An exhaustive evaluation using tailor-made data sets (ProSPECCTs). PLOS Computational Biology 2018, 14 (11), e1006483 10.1371/journal.pcbi.1006483. 30408032 PMC6224041

[ref71] Ehrt C.; Brinkjost T.; Koch O. Impact of Binding Site Comparisons on Medicinal Chemistry and Rational Molecular Design. J. Med. Chem. 2016, 59 (9), 4121–4151. 10.1021/acs.jmedchem.6b00078. 27046190

[ref72] Clark J. J.; Orban Z. J.; Carlson H. A. Predicting binding sites from unbound versus bound protein structures. Sci. Rep 2020, 10 (1), 15856 (acccessed 2023/06/11/20:18:15). From www.nature.com 10.1038/s41598-020-72906-7. 32985584 PMC7522209

[ref73] Binkowski T. A.; Naghibzadeh S.; Liang J. CASTp: Computed Atlas of Surface Topography of proteins. Nucleic Acids Res. 2003, 31 (13), 3352–3355. (acccessed 7/9/2023) 10.1093/nar/gkg512. 12824325 PMC168919

[ref74] Liang J.; Woodward C.; Edelsbrunner H. Anatomy of protein pockets and cavities: Measurement of binding site geometry and implications for ligand design. Protein Sci. 1998, 7 (9), 1884–1897. (acccessed 2023/07/09) 10.1002/pro.5560070905. 9761470 PMC2144175

[ref75] Hendlich M.; Rippmann F.; Barnickel G. LIGSITE: automatic and efficient detection of potential small molecule-binding sites in proteins. Journal of Molecular Graphics and Modelling 1997, 15 (6), 359–363. 10.1016/S1093-3263(98)00002-3. 9704298

[ref76] Laurie A. T. R.; Jackson R. M. Q-SiteFinder: an energy-based method for the prediction of protein-ligand binding sites. Bioinformatics 2005, 21 (9), 1908–1916. (acccessed 7/9/2023) 10.1093/bioinformatics/bti315. 15701681

[ref77] Hernandez M.; Ghersi D.; Sanchez R. SITEHOUND-web: a server for ligand binding site identification in protein structures. Nucleic acids research 2009, 37 (suppl_2), W413–W416. 10.1093/nar/gkp281. 19398430 PMC2703923

[ref78] Krivák R.; Hoksza D. P2Rank: machine learning based tool for rapid and accurate prediction of ligand binding sites from protein structure. Journal of Cheminformatics 2018, 10 (1), 39 10.1186/s13321-018-0285-8. 30109435 PMC6091426

[ref79] Ngan C.-H.; Hall D. R.; Zerbe B.; Grove L. E.; Kozakov D.; Vajda S. FTSite: high accuracy detection of ligand binding sites on unbound protein structures. Bioinformatics 2012, 28 (2), 286–287. 10.1093/bioinformatics/btr651. 22113084 PMC3259439

[ref80] Liao J.; Wang Q.; Wu F.; Huang Z. In Silico Methods for Identification of Potential Active Sites of Therapeutic Targets. Molecules 2022, 27, 7103 10.3390/molecules27207103. 36296697 PMC9609013

[ref81] Roche D. B.; Brackenridge D. A.; McGuffin L. J. Proteins and Their Interacting Partners: An Introduction to Protein-Ligand Binding Site Prediction Methods. International Journal of Molecular Sciences 2015, 16, 29829–29842. 10.3390/ijms161226202. 26694353 PMC4691145

[ref82] Di Palma F.; Abate C.; Decherchi S.; Cavalli A. Ligandability and druggability assessment via machine learning. WIREs Computational Molecular Science 2023, 13, e1676 (acccessed 2023/07/10) 10.1002/wcms.1676.

[ref83] Gagliardi L.; Raffo A.; Fugacci U.; Biasotti S.; Rocchia W.; Huang H.; Amor B. B.; Fang Y.; Zhang Y.; Wang X.; et al. SHREC 2022: Protein-ligand binding site recognition. Computers & Graphics 2022, 107, 20–31. 10.1016/j.cag.2022.07.005.

[ref84] Schmidtke P.; Souaille C.; Estienne F.; Baurin N.; Kroemer R. T. Large-Scale Comparison of Four Binding Site Detection Algorithms. J. Chem. Inf. Model. 2010, 50 (12), 2191–2200. 10.1021/ci1000289. 20828173

[ref85] Richards F. M. The interpretation of protein structures: total volume, group volume distributions and packing density. Journal of molecular biology 1974, 82 (1), 1–14. 10.1016/0022-2836(74)90570-1. 4818482

[ref86] Ccgi M. Molecular operating environment (MOE), 2013.08. Chemical Computing Group Inc., Montreal 2016, 354.

[ref87] Aggarwal R.; Gupta A.; Chelur V.; Jawahar C. V.; Priyakumar U. D. DeepPocket: Ligand Binding Site Detection and Segmentation using 3D Convolutional Neural Networks. J. Chem. Inf. Model. 2022, 62 (21), 5069–5079. 10.1021/acs.jcim.1c00799. 34374539

[ref88] Özçelik R.; van Tilborg D.; Jiménez-Luna J.; Grisoni F. Structure-Based Drug Discovery with Deep Learning**. ChemBioChem. 2023, 24 (13), e202200776 (acccessed 2023/07/17) 10.1002/cbic.202200776. 37014633

[ref89] Mylonas S. K.; Axenopoulos A.; Daras P. DeepSurf: a surface-based deep learning approach for the prediction of ligand binding sites on proteins. Bioinformatics 2021, 37 (12), 1681–1690. (acccessed 7/10/2023) 10.1093/bioinformatics/btab009. 33471069

[ref90] Gainza P.; Sverrisson F.; Monti F.; Rodolà E.; Boscaini D.; Bronstein M. M.; Correia B. E. Deciphering interaction fingerprints from protein molecular surfaces using geometric deep learning. Nat. Methods 2020, 17 (2), 184–192. 10.1038/s41592-019-0666-6. 31819266

[ref91] Sverrisson F.; Feydy J.; Correia B. E.; Bronstein M. M. Fast end-to-end learning on protein surfaces. bioRxiv: 2020.

[ref92] Smith Z.; Strobel M.; Vani B. P.; Tiwary P. Graph Attention Site Prediction (GrASP): Identifying Druggable Binding Sites Using Graph Neural Networks with Attention. bioRxiv 2023, 10.1101/2023.07.25.550565. PMC11182664 38453912

[ref93] Rooklin D.; Modell A. E.; Li H.; Berdan V.; Arora P. S.; Zhang Y. Targeting Unoccupied Surfaces on Protein-Protein Interfaces. J. Am. Chem. Soc. 2017, 139 (44), 15560–15563. (acccessed 2023/06/11/21:32:54). From ACS Publications 10.1021/jacs.7b05960. 28759230 PMC5677581

[ref94] Sadek J.; Wuo M. G.; Rooklin D.; Hauenstein A.; Hong S. H.; Gautam A.; Wu H.; Zhang Y.; Cesarman E.; Arora P. S. Modulation of virus-induced NF-κB signaling by NEMO coiled coil mimics. Nat. Commun. 2020, 11 (1), 1786 (acccessed 2023/06/11/21:28:12). From www.nature.com 10.1038/s41467-020-15576-3. 32286300 PMC7156456

[ref95] Torner J. M.; Yang Y.; Rooklin D.; Zhang Y.; Arora P. S. Identification of Secondary Binding Sites on Protein Surfaces for Rational Elaboration of Synthetic Protein Mimics. ACS Chem. Biol. 2021, 16 (7), 1179–1183. (acccessed 2023/06/11/20:40:38). From ACS Publications 10.1021/acschembio.1c00418. 34228913 PMC8344045

[ref96] Modell A. E.; Marrone F. III; Panigrahi N. R.; Zhang Y.; Arora P. S. Peptide Tethering: Pocket-Directed Fragment Screening for Peptidomimetic Inhibitor Discovery. J. Am. Chem. Soc. 2022, 144 (3), 1198–1204. (acccessed 2023/06/11/21:20:39). From ACS Publications 10.1021/jacs.1c09666. 35029987 PMC8959088

[ref97] Wu Y.; Gao X.-Y.; Chen X.-H.; Zhang S.-L.; Wang W.-J.; Sheng X.-H.; Chen D.-Z. Fragment-centric topographic mapping method guides the understanding of ABCG2-inhibitor interactions. RSC Adv. 2019, 9 (14), 7757–7766. (acccessed 2023/06/11/21:43:13). From pubs.rsc.org 10.1039/C8RA09789E. 35521159 PMC9061187

[ref98] Li W.-J.; Chen X.-H.; Zeng J.-C.; Duan L.-L.; Liu Z.-H.; Sheng X.-H. Theoretical insight into the multiple interactions of quinazoline inhibitors with breast cancer resistance protein (BCRP/ABCG2). J. Biomol. Struct. Dyn. 2020, 38 (14), 4336–4343. 10.1080/07391102.2019.1677503. 31594454

[ref99] Hou X.; Sun J.-p.; Ge L.; Liang X.; Li K.; Zhang Y.; Fang H. Inhibition of striatal-enriched protein tyrosine phosphatase by targeting computationally revealed cryptic pockets. Eur. J. Med. Chem. 2020, 190, 112131 (acccessed 2023/06/11/21:37:08). From ScienceDirect 10.1016/j.ejmech.2020.112131. 32078861 PMC7163917

[ref100] Kawai K.; Nagata N.; Takahashi Y. De Novo Design of Drug-Like Molecules by a Fragment-Based Molecular Evolutionary Approach. J. Chem. Inf. Model. 2014, 54 (1), 49–56. 10.1021/ci400418c. 24372539

[ref101] Lionta E.; Spyrou G.; Vassilatis D.; Cournia Z. Structure-based virtual screening for drug discovery: principles, applications and recent advances. Current topics in medicinal chemistry 2014, 14 (16), 1923–1938. 10.2174/1568026614666140929124445. 25262799 PMC4443793

[ref102] Gorgulla C.; Boeszoermenyi A.; Wang Z.-F.; Fischer P. D.; Coote P. W.; Padmanabha Das K. M.; Malets Y. S.; Radchenko D. S.; Moroz Y. S.; Scott D. A.; et al. An open-source drug discovery platform enables ultra-large virtual screens. Nature 2020, 580 (7805), 663–668. 10.1038/s41586-020-2117-z. 32152607 PMC8352709

[ref103] Stumpfe D.; Bajorath J. Current Trends, Overlooked Issues, and Unmet Challenges in Virtual Screening. J. Chem. Inf. Model. 2020, 60 (9), 4112–4115. 10.1021/acs.jcim.9b01101. 32011879

[ref104] Talele T. T.; Khedkar S. A.; Rigby A. C. Successful applications of computer aided drug discovery: moving drugs from concept to the clinic. Current topics in medicinal chemistry 2010, 10 (1), 127–141. 10.2174/156802610790232251. 19929824

[ref105] Stein R. M.; Kang H. J.; McCorvy J. D.; Glatfelter G. C.; Jones A. J.; Che T.; Slocum S.; Huang X.-P.; Savych O.; Moroz Y. S.; et al. Virtual discovery of melatonin receptor ligands to modulate circadian rhythms. Nature 2020, 579 (7800), 609–614. 10.1038/s41586-020-2027-0. 32040955 PMC7134359

[ref106] Van Drie J. H. Computer-aided drug design: the next 20 years. Journal of Computer-Aided Molecular Design 2007, 21 (10), 591–601. 10.1007/s10822-007-9142-y. 17989929

[ref107] Yang C.; Chen E. A.; Zhang Y. Protein-Ligand Docking in the Machine-Learning Era. Molecules 2022, 27 (14), 4568 10.3390/molecules27144568. 35889440 PMC9323102

[ref108] Hartman G. D.; Egbertson M. S.; Halczenko W.; Laswell W. L.; Duggan M. E.; Smith R. L.; Naylor A. M.; Manno P. D.; Lynch R. J. Non-peptide fibrinogen receptor antagonists. 1. Discovery and design of exosite inhibitors. J. Med. Chem. 1992, 35 (24), 4640–4642. 10.1021/jm00102a020. 1469694

[ref109] Greer J.; Erickson J. W.; Baldwin J. J.; Varney M. D. Application of the Three-Dimensional Structures of Protein Target Molecules in Structure-Based Drug Design. J. Med. Chem. 1994, 37 (8), 1035–1054. 10.1021/jm00034a001. 8164249

[ref110] Wlodawer A.; Vondrasek J. INHIBITORS OF HIV-1 PROTEASE: A Major Success of Structure-Assisted Drug Design. Annu. Rev. Biophys. Biomol. Struct. 1998, 27 (1), 249–284. (acccessed 2023/06/22) 10.1146/annurev.biophys.27.1.249. 9646869

[ref111] Jhoti H.; Leach A. R. Structure-based drug discovery; Springer: 2007.

[ref112] Lavecchia A.; Di Giovanni C. Virtual screening strategies in drug discovery: a critical review. Curr. Med. Chem. 2013, 20 (23), 2839–2860. 10.2174/09298673113209990001. 23651302

[ref113] Reddy A. S.; Pati S. P.; Kumar P. P.; Pradeep H.; Sastry G. N. Virtual screening in drug discovery-a computational perspective. Current Protein and Peptide Science 2007, 8 (4), 329–351. 10.2174/138920307781369427. 17696867

[ref114] Ferreira L. G.; Dos Santos R. N.; Oliva G.; Andricopulo A. D. Molecular Docking and Structure-Based Drug Design Strategies. Molecules 2015, 20 (7), 13384–13421. 10.3390/molecules200713384. 26205061 PMC6332083

[ref115] De Ruyck J.; Brysbaert G.; Blossey R.; Lensink M. F. Molecular docking as a popular tool in drug design, an in silico travel. Advances and Applications in Bioinformatics and Chemistry 2016, Volume 9, 1–11. 10.2147/AABC.S105289. PMC4930227 27390530

[ref116] Torres P. H. M.; Sodero A. C. R.; Jofily P.; Silva-Jr F. P. Key Topics in Molecular Docking for Drug Design. International Journal of Molecular Sciences 2019, 20 (18), 4574 10.3390/ijms20184574. 31540192 PMC6769580

[ref117] Fan J.; Fu A.; Zhang L. Progress in molecular docking. Quantitative Biology 2019, 7, 83–89. 10.1007/s40484-019-0172-y.

[ref118] Koshland Jr D. E. The key-lock theory and the induced fit theory. Angewandte Chemie International Edition in English 1995, 33 (23–24), 2375–2378. 10.1002/anie.199423751.

[ref119] Allen W. J.; Balius T. E.; Mukherjee S.; Brozell S. R.; Moustakas D. T.; Lang P. T.; Case D. A.; Kuntz I. D.; Rizzo R. C. DOCK 6: Impact of new features and current docking performance. Journal of computational chemistry 2015, 36 (15), 1132–1156. 10.1002/jcc.23905. 25914306 PMC4469538

[ref120] Halgren T. A.; Murphy R. B.; Friesner R. A.; Beard H. S.; Frye L. L.; Pollard W. T.; Banks J. L. Glide: A New Approach for Rapid, Accurate Docking and Scoring. 2. Enrichment Factors in Database Screening. J. Med. Chem. 2004, 47 (7), 1750–1759. 10.1021/jm030644s. 15027866

[ref121] Goodsell D. S.; Morris G. M.; Olson A. J. Automated docking of flexible ligands: applications of AutoDock. Journal of molecular recognition 1996, 9 (1), 1–5. 10.1002/(SICI)1099-1352(199601)9:1<1::AID-JMR241>3.0.CO;2-6. 8723313

[ref122] Jorgensen W. L.; Thomas L. L. Perspective on Free-Energy Perturbation Calculations for Chemical Equilibria. J. Chem. Theory Comput. 2008, 4 (6), 869–876. 10.1021/ct800011m. 19936324 PMC2779535

[ref123] Deflorian F.; Perez-Benito L.; Lenselink E. B.; Congreve M.; van Vlijmen H. W. T.; Mason J. S.; Graaf C. d.; Tresadern G. Accurate Prediction of GPCR Ligand Binding Affinity with Free Energy Perturbation. J. Chem. Inf. Model. 2020, 60 (11), 5563–5579. 10.1021/acs.jcim.0c00449. 32539374

[ref124] Bhati A. P.; Wan S.; Wright D. W.; Coveney P. V. Rapid, Accurate, Precise, and Reliable Relative Free Energy Prediction Using Ensemble Based Thermodynamic Integration. J. Chem. Theory Comput. 2017, 13 (1), 210–222. 10.1021/acs.jctc.6b00979. 27997169

[ref125] Genheden S.; Nilsson I.; Ryde U. Binding Affinities of Factor Xa Inhibitors Estimated by Thermodynamic Integration and MM/GBSA. J. Chem. Inf. Model. 2011, 51 (4), 947–958. 10.1021/ci100458f. 21417269

[ref126] Li J.; Fu A.; Zhang L. An Overview of Scoring Functions Used for Protein-Ligand Interactions in Molecular Docking. Interdisciplinary Sciences: Computational Life Sciences 2019, 11 (2), 320–328. 10.1007/s12539-019-00327-w. 30877639

[ref127] Böhm H. J.; Stahl M. The use of scoring functions in drug discovery applications. Reviews in computational chemistry 2002, 18, 41–87. 10.1002/0471433519.ch2.

[ref128] Huang S.-Y.; Grinter S. Z.; Zou X. Scoring functions and their evaluation methods for protein-ligand docking: recent advances and future directions. Phys. Chem. Chem. Phys. 2010, 12 (40), 12899–12908. 10.1039/c0cp00151a. 20730182 PMC11103779

[ref129] Lyu J.; Wang S.; Balius T. E.; Singh I.; Levit A.; Moroz Y. S.; O’Meara M. J.; Che T.; Algaa E.; Tolmachova K.; et al. Ultra-large library docking for discovering new chemotypes. Nature 2019, 566 (7743), 224–229. 10.1038/s41586-019-0917-9. 30728502 PMC6383769

[ref130] Talele T. T.; Arora P.; Kulkarni S. S.; Patel M. R.; Singh S.; Chudayeu M.; Kaushik-Basu N. Structure-based virtual screening, synthesis and SAR of novel inhibitors of hepatitis C virus NS5B polymerase. Bioorg. Med. Chem. 2010, 18 (13), 4630–4638. 10.1016/j.bmc.2010.05.030. 20627595 PMC2956004

[ref131] Su M.; Yang Q.; Du Y.; Feng G.; Liu Z.; Li Y.; Wang R. Comparative Assessment of Scoring Functions: The CASF-2016 Update. J. Chem. Inf. Model. 2019, 59 (2), 895–913. 10.1021/acs.jcim.8b00545. 30481020

[ref132] Gohlke H.; Hendlich M.; Klebe G. Knowledge-based scoring function to predict protein-ligand interactions11Edited by R. Huber. J. Mol. Biol. 2000, 295 (2), 337–356. 10.1006/jmbi.1999.3371. 10623530

[ref133] Huang S. Y.; Zou X. An iterative knowledge-based scoring function to predict protein-ligand interactions: I. Derivation of interaction potentials. Journal of computational chemistry 2006, 27 (15), 1866–1875. 10.1002/jcc.20504. 16983673

[ref134] Huang S. Y.; Zou X. An iterative knowledge-based scoring function to predict protein-ligand interactions: II. Validation of the scoring function. Journal of computational chemistry 2006, 27 (15), 1876–1882. 10.1002/jcc.20505. 16983671

[ref135] Muegge I.; Martin Y. C. A General and Fast Scoring Function for Protein-Ligand Interactions: A Simplified Potential Approach. J. Med. Chem. 1999, 42 (5), 791–804. 10.1021/jm980536j. 10072678

[ref136] Böhm H. J. A novel computational tool for automated structure-based drug design. Journal of Molecular Recognition 1993, 6 (3), 131–137. 10.1002/jmr.300060305. 8060670

[ref137] Böhm H.-J. The development of a simple empirical scoring function to estimate the binding constant for a protein-ligand complex of known three-dimensional structure. Journal of Computer-Aided Molecular Design 1994, 8 (3), 243–256. 10.1007/BF00126743. 7964925

[ref138] Wang R.; Lai L.; Wang S. Further development and validation of empirical scoring functions for structure-based binding affinity prediction. Journal of Computer-Aided Molecular Design 2002, 16 (1), 11–26. 10.1023/A:1016357811882. 12197663

[ref139] Yang C.; Zhang Y. Lin_F9: A Linear Empirical Scoring Function for Protein-Ligand Docking. J. Chem. Inf. Model. 2021, 61 (9), 4630–4644. 10.1021/acs.jcim.1c00737. 34469692 PMC8478859

[ref140] Ain Q. U.; Aleksandrova A.; Roessler F. D.; Ballester P. J. Machine-learning scoring functions to improve structure-based binding affinity prediction and virtual screening. Wiley Interdisciplinary Reviews: Computational Molecular Science 2015, 5 (6), 405–424. 10.1002/wcms.1225. 27110292 PMC4832270

[ref141] Li H.; Sze K. H.; Lu G.; Ballester P. J. Machine-learning scoring functions for structure-based virtual screening. Wiley Interdisciplinary Reviews: Computational Molecular Science 2021, 11 (1), e1478 10.1002/wcms.1478. PMC4832270 27110292

[ref142] Cortes C.; Vapnik V. Support-vector networks. Machine Learning 1995, 20 (3), 273–297. 10.1007/BF00994018.

[ref143] Liaw A.; Wiener M. Classification and regression by randomForest. R news 2002, 2 (3), 18–22.

[ref144] Chen T.; Guestrin C. Xgboost: A scalable tree boosting system. In Proceedings of the 22nd acm sigkdd international conference on knowledge discovery and data mining, 2016; pp 785–794.

[ref145] Ballester P. J.; Mitchell J. B. O. A machine learning approach to predicting protein-ligand binding affinity with applications to molecular docking. Bioinformatics 2010, 26 (9), 1169–1175. (acccessed 6/24/2023) 10.1093/bioinformatics/btq112. 20236947 PMC3524828

[ref146] Zilian D.; Sotriffer C. A. SFCscoreRF: A Random Forest-Based Scoring Function for Improved Affinity Prediction of Protein-Ligand Complexes. J. Chem. Inf. Model. 2013, 53 (8), 1923–1933. 10.1021/ci400120b. 23705795

[ref147] Ashtawy H. M.; Mahapatra N. R. Task-Specific Scoring Functions for Predicting Ligand Binding Poses and Affinity and for Screening Enrichment. J. Chem. Inf. Model. 2018, 58 (1), 119–133. 10.1021/acs.jcim.7b00309. 29190087

[ref148] Wójcikowski M.; Ballester P. J.; Siedlecki P. Performance of machine-learning scoring functions in structure-based virtual screening. Sci. Rep 2017, 7 (1), 46710 10.1038/srep46710. 28440302 PMC5404222

[ref149] Wang C.; Zhang Y. Improving scoring-docking-screening powers of protein-ligand scoring functions using random forest. Journal of computational chemistry 2017, 38 (3), 169–177. 10.1002/jcc.24667. 27859414 PMC5140681

[ref150] Li Y.; Han L.; Liu Z.; Wang R. Comparative Assessment of Scoring Functions on an Updated Benchmark: 2. Evaluation Methods and General Results. J. Chem. Inf. Model. 2014, 54 (6), 1717–1736. 10.1021/ci500081m. 24708446

[ref151] Cheng T.; Li X.; Li Y.; Liu Z.; Wang R. Comparative assessment of scoring functions on a diverse test set. J. Chem. Inf. Model. 2009, 49 (4), 1079–1093. 10.1021/ci9000053. 19358517

[ref152] Lu J.; Hou X.; Wang C.; Zhang Y. Incorporating Explicit Water Molecules and Ligand Conformation Stability in Machine-Learning Scoring Functions. J. Chem. Inf. Model. 2019, 59 (11), 4540–4549. 10.1021/acs.jcim.9b00645. 31638801 PMC6878146

[ref153] Chen T.; Guestrin C. XGBoost: A Scalable Tree Boosting System. In Proceedings of the 22nd ACM SIGKDD International Conference on Knowledge Discovery and Data Mining, San Francisco, California, USA; 2016.

[ref154] Yang C.; Zhang Y. Delta Machine Learning to Improve Scoring-Ranking-Screening Performances of Protein-Ligand Scoring Functions. J. Chem. Inf. Model. 2022, 62 (11), 2696–2712. 10.1021/acs.jcim.2c00485. 35579568 PMC9197983

[ref155] Tran-Nguyen V.-K.; Jacquemard C.; Rognan D. LIT-PCBA: An Unbiased Data Set for Machine Learning and Virtual Screening. J. Chem. Inf. Model. 2020, 60 (9), 4263–4273. 10.1021/acs.jcim.0c00155. 32282202

[ref156] Tran-Nguyen V.-K.; Bret G.; Rognan D. True accuracy of fast scoring functions to predict High-Throughput screening data from docking poses: The simpler the better. J. Chem. Inf. Model. 2021, 61 (6), 2788–2797. 10.1021/acs.jcim.1c00292. 34109796

[ref157] Durrant J. D.; McCammon J. A. NNScore: A Neural-Network-Based Scoring Function for the Characterization of Protein-Ligand Complexes. J. Chem. Inf. Model. 2010, 50 (10), 1865–1871. 10.1021/ci100244v. 20845954 PMC2964041

[ref158] Durrant J. D.; McCammon J. A. NNScore 2.0: A Neural-Network Receptor-Ligand Scoring Function. J. Chem. Inf. Model. 2011, 51 (11), 2897–2903. 10.1021/ci2003889. 22017367 PMC3225089

[ref159] Wallach I.; Dzamba M.; Heifets A. AtomNet: a deep convolutional neural network for bioactivity prediction in structure-based drug discovery. arXiv preprint arXiv:1510.02855 2015.

[ref160] Ragoza M.; Hochuli J.; Idrobo E.; Sunseri J.; Koes D. R. Protein-Ligand Scoring with Convolutional Neural Networks. J. Chem. Inf. Model. 2017, 57 (4), 942–957. 10.1021/acs.jcim.6b00740. 28368587 PMC5479431

[ref161] Stepniewska-Dziubinska M. M.; Zielenkiewicz P.; Siedlecki P. Development and evaluation of a deep learning model for protein-ligand binding affinity prediction. Bioinformatics 2018, 34 (21), 3666–3674. (acccessed 6/29/2023) 10.1093/bioinformatics/bty374. 29757353 PMC6198856

[ref162] Jiménez J.; Škalič M.; Martínez-Rosell G.; De Fabritiis G. KDEEP: Protein-Ligand Absolute Binding Affinity Prediction via 3D-Convolutional Neural Networks. J. Chem. Inf. Model. 2018, 58 (2), 287–296. 10.1021/acs.jcim.7b00650. 29309725

[ref163] Zheng L.; Fan J.; Mu Y. OnionNet: a Multiple-Layer Intermolecular-Contact-Based Convolutional Neural Network for Protein-Ligand Binding Affinity Prediction. ACS Omega 2019, 4 (14), 15956–15965. 10.1021/acsomega.9b01997. 31592466 PMC6776976

[ref164] Wang Z.; Zheng L.; Liu Y.; Qu Y.; Li Y.-Q.; Zhao M.; Mu Y.; Li W. OnionNet-2: a convolutional neural network model for predicting protein-ligand binding affinity based on residue-atom contacting shells. Frontiers in Chemistry 2021, 9, 913 10.3389/fchem.2021.753002. PMC8579074 34778208

[ref165] Feinberg E. N.; Sur D.; Wu Z.; Husic B. E.; Mai H.; Li Y.; Sun S.; Yang J.; Ramsundar B.; Pande V. S. PotentialNet for Molecular Property Prediction. ACS Central Science 2018, 4 (11), 1520–1530. 10.1021/acscentsci.8b00507. 30555904 PMC6276035

[ref166] Lim J.; Ryu S.; Park K.; Choe Y. J.; Ham J.; Kim W. Y. Predicting Drug-Target Interaction Using a Novel Graph Neural Network with 3D Structure-Embedded Graph Representation. J. Chem. Inf. Model. 2019, 59 (9), 3981–3988. 10.1021/acs.jcim.9b00387. 31443612

[ref167] Karlov D. S.; Sosnin S.; Fedorov M. V.; Popov P. graphDelta: MPNN Scoring Function for the Affinity Prediction of Protein-Ligand Complexes. ACS Omega 2020, 5 (10), 5150–5159. 10.1021/acsomega.9b04162. 32201802 PMC7081425

[ref168] Li S.; Zhou J.; Xu T.; Huang L.; Wang F.; Xiong H.; Huang W.; Dou D.; Xiong H. Structure-aware interactive graph neural networks for the prediction of protein-ligand binding affinity. In Proceedings of the 27th ACM SIGKDD Conference on Knowledge Discovery & Data Mining, 2021; pp 975–985.

[ref169] Son J.; Kim D. Development of a graph convolutional neural network model for efficient prediction of protein-ligand binding affinities. PloS one 2021, 16 (4), e0249404 10.1371/journal.pone.0249404. 33831016 PMC8031450

[ref170] Méndez-Lucio O.; Ahmad M.; del Rio-Chanona E. A.; Wegner J. K. A geometric deep learning approach to predict binding conformations of bioactive molecules. Nature Machine Intelligence 2021, 3 (12), 1033–1039. 10.1038/s42256-021-00409-9.

[ref171] Shen C.; Zhang X.; Deng Y.; Gao J.; Wang D.; Xu L.; Pan P.; Hou T.; Kang Y. Boosting Protein-Ligand Binding Pose Prediction and Virtual Screening Based on Residue-Atom Distance Likelihood Potential and Graph Transformer. J. Med. Chem. 2022, 65 (15), 10691–10706. 10.1021/acs.jmedchem.2c00991. 35917397

[ref172] Moon S.; Zhung W.; Yang S.; Lim J.; Kim W. Y. PIGNet: a physics-informed deep learning model toward generalized drug-target interaction predictions. Chemical Science 2022, 13 (13), 3661–3673. 10.1039/D1SC06946B. 35432900 PMC8966633

[ref173] Bao J.; He X.; Zhang J. Z. H. DeepBSP—a Machine Learning Method for Accurate Prediction of Protein-Ligand Docking Structures. J. Chem. Inf. Model. 2021, 61 (5), 2231–2240. 10.1021/acs.jcim.1c00334. 33979150

[ref174] Zheng L.; Meng J.; Jiang K.; Lan H.; Wang Z.; Lin M.; Li W.; Guo H.; Wei Y.; Mu Y. Improving protein-ligand docking and screening accuracies by incorporating a scoring function correction term. Briefings in Bioinformatics 2022, 23 ( (3), ). 10.1093/bib/bbac051 (acccessed 7/16/2023). PMC9116214 35289359

[ref175] Kadukova M.; Machado K. d. S.; Chacón P.; Grudinin S. KORP-PL: a coarse-grained knowledge-based scoring function for protein-ligand interactions. Bioinformatics 2021, 37 (7), 943–950. 10.1093/bioinformatics/btaa748. 32840574

[ref176] Korb O.; Stützle T.; Exner T. E. Empirical Scoring Functions for Advanced Protein-Ligand Docking with PLANTS. J. Chem. Inf. Model. 2009, 49 (1), 84–96. 10.1021/ci800298z. 19125657

[ref177] Hou T.; Shen C.; Zhang X.; Hsieh C.-Y.; Deng Y.; Wang D.; Xu L.; Wu J.; Li D.; Kang Y. A generalized protein-ligand scoring framework with balanced scoring, docking, ranking and screening powers. Chemical Science 2023, 14, 8129 10.1039/D3SC02044D. 37538816 PMC10395315

[ref178] Stärk H.; Ganea O.; Pattanaik L.; Barzilay D. R.; Jaakkola T. EquiBind: Geometric Deep Learning for Drug Binding Structure Prediction. In Proceedings of the 39th International Conference on Machine Learning, Proceedings of Machine Learning Research; 2022.

[ref179] Lu W.; Wu Q.; Zhang J.; Rao J.; Li C.; Zheng S. TANKBind: Trigonometry-Aware Neural NetworKs for Drug-Protein Binding Structure Prediction. bioRxiv 2022, 2022.2006.2006.495043 10.1101/2022.06.06.495043.

[ref180] Corso G.; Stärk H.; Jing B.; Barzilay R.; Jaakkola T. Diffdock: Diffusion steps, twists, and turns for molecular docking. arXiv preprint arXiv:2210.01776 2022.

[ref181] Rossi M.; Chutia S.; Scheffler M.; Blum V. Validation Challenge of Density-Functional Theory for Peptides—Example of Ac-Phe-Ala5-LysH+. J. Phys. Chem. A 2014, 118 (35), 7349–7359. 10.1021/jp412055r. 24405171

[ref182] Hawkins P. C. D. Conformation Generation: The State of the Art. J. Chem. Inf. Model. 2017, 57 (8), 1747–1756. 10.1021/acs.jcim.7b00221. 28682617

[ref183] Halgren T. A. Merck molecular force field. II. MMFF94 van der Waals and electrostatic parameters for intermolecular interactions. J. Comput. Chem. 1996, 17 (5–6), 520–552. 10.1002/(SICI)1096-987X(199604)17:5/6<520::AID-JCC2>3.0.CO;2-W.

[ref184] Bannwarth C.; Ehlert S.; Grimme S. GFN2-xTB—An Accurate and Broadly Parametrized Self-Consistent Tight-Binding Quantum Chemical Method with Multipole Electrostatics and Density-Dependent Dispersion Contributions. J. Chem. Theory Comput. 2019, 15 (3), 1652–1671. 10.1021/acs.jctc.8b01176. 30741547

[ref185] Keith J. A.; Vassilev-Galindo V.; Cheng B.; Chmiela S.; Gastegger M.; Müller K.-R.; Tkatchenko A. Combining Machine Learning and Computational Chemistry for Predictive Insights Into Chemical Systems. Chem. Rev. 2021, 121 (16), 9816–9872. 10.1021/acs.chemrev.1c00107. 34232033 PMC8391798

[ref186] von Lilienfeld O. A.; Muller K.-R.; Tkatchenko A. Exploring chemical compound space with quantum-based machine learning. Nature Reviews Chemistry 2020, 4 (7), 347–358. 10.1038/s41570-020-0189-9. 37127950

[ref187] Musil F.; Grisafi A.; Bartók A. P.; Ortner C.; Csányi G. b.; Ceriotti M. Physics-Inspired Structural Representations for Molecules and Materials. Chem. Rev. 2021, 121 (16), 9759–9815. 10.1021/acs.chemrev.1c00021. 34310133

[ref188] Langer M. F.; Goeßmann A.; Rupp M. Representations of molecules and materials for interpolation of quantum-mechanical simulations via machine learning. npj Computational Materials 2022, 8 (1), 41 10.1038/s41524-022-00721-x.

[ref189] Haghighatlari M.; Li J.; Heidar-Zadeh F.; Liu Y.; Guan X.; Head-Gordon T. Learning to Make Chemical Predictions: The Interplay of Feature Representation, Data, and Machine Learning Methods. Chem. 2020, 6 (7), 1527–1542. 10.1016/j.chempr.2020.05.014. 32695924 PMC7373218

[ref190] Huang B.; von Lilienfeld O. A. Ab Initio Machine Learning in Chemical Compound Space. Chem. Rev. 2021, 121 (16), 10001–10036. 10.1021/acs.chemrev.0c01303. 34387476 PMC8391942

[ref191] Dral P. O. Quantum Chemistry in the Age of Machine Learning. J. Phys. Chem. Lett. 2020, 11 (6), 2336–2347. 10.1021/acs.jpclett.9b03664. 32125858

[ref192] LeCun Y.; Bengio Y.; Hinton G. Deep learning. Nature 2015, 521 (7553), 436–444. 10.1038/nature14539. 26017442

[ref193] Rupp M.; Tkatchenko A.; Muller K.-R.; von Lilienfeld O. A. v. Fast and Accurate Modeling of Molecular Atomization Energies with Machine Learning. Phys. Rev. Lett. 2012, 108 (5), 058301 10.1103/PhysRevLett.108.058301. 22400967

[ref194] Montavon G.; Rupp M.; Gobre V.; Vazquez-Mayagoitia A.; Hansen K.; Tkatchenko A.; Müller K.-R.; Lilienfeld O. A. v. Machine learning of molecular electronic properties in chemical compound space. New J. Phys. 2013, 15 (9), 095003 10.1088/1367-2630/15/9/095003.

[ref195] Hansen K.; Montavon G.; Biegler F.; Fazli S.; Rupp M.; Scheffler M.; Lilienfeld O. A. v.; Tkatchenko A.; Müller K.-R. Assessment and Validation of Machine Learning Methods for Predicting Molecular Atomization Energies. J. Chem. Theory Comput. 2013, 9 (8), 3404–3419. 10.1021/ct400195d. 26584096

[ref196] Hirn M.; Poilvert N.; Mallat S. Quantum Energy Regression using Scattering Transforms. arXiv:1502.02077 2015.

[ref197] Hansen K.; Biegler F.; Ramakrishnan R.; Pronobis W.; von Lilienfeld O. A.; Müller K.-R.; Tkatchenko A. Machine Learning Predictions of Molecular Properties: Accurate Many-Body Potentials and Nonlocality in Chemical Space. J. Phys. Chem. Lett. 2015, 6 (12), 2326–2331. 10.1021/acs.jpclett.5b00831. 26113956 PMC4476293

[ref198] Bartók A. P.; Payne M. C.; Kondor R.; Csányi G. Gaussian Approximation Potentials: The Accuracy of Quantum Mechanics, without the Electrons. Phys. Rev. Lett. 2010, 104 (13), 136403 10.1103/PhysRevLett.104.136403. 20481899

[ref199] Bartók A. P.; Kondor R.; Csányi G. On representing chemical environments. Phys. Rev. B 2013, 87 (18), 184115 10.1103/PhysRevB.87.184115.

[ref200] Behler J. Atom-centered symmetry functions for constructing high-dimensional neural network potentials. J. Chem. Phys. 2011, 134 (7), 074106 10.1063/1.3553717. 21341827

[ref201] Behler J. Neural network potential-energy surfaces in chemistry: a tool for large-scale simulations. Phys. Chem. Chem. Phys. 2011, 13 (40), 17930–17955. 10.1039/c1cp21668f. 21915403

[ref202] Behler J. Constructing high-dimensional neural network potentials: A tutorial review. Int. J. Quantum Chem. 2015, 115 (16), 1032–1050. 10.1002/qua.24890.

[ref203] Smith J. S.; Isayev O.; Roitberg A. E. ANI-1: an extensible neural network potential with DFT accuracy at force field computational cost. Chemical Science 2017, 8 (4), 3192–3203. 10.1039/C6SC05720A. 28507695 PMC5414547

[ref204] Schütt K. T.; Arbabzadah F.; Chmiela S.; Müller K. R.; Tkatchenko A. Quantum-chemical insights from deep tensor neural networks. Nat. Commun. 2017, 8 (1), 13890 10.1038/ncomms13890. 28067221 PMC5228054

[ref205] Gilmer J.; Schoenholz S. S.; Riley P. F.; Vinyals O.; Dahl G. E. Neural Message Passing for Quantum Chemistry. In Proceedings of the 34th International Conference on Machine Learning, Proceedings of Machine Learning Research; 2017.

[ref206] Schütt K. T.; Sauceda H. E.; Kindermans P. J.; Tkatchenko A.; Müller K. R. SchNet - A deep learning architecture for molecules and materials. J. Chem. Phys. 2018, 148 (24), 241722 10.1063/1.5019779. 29960322

[ref207] Lubbers N.; Smith J. S.; Barros K. Hierarchical modeling of molecular energies using a deep neural network. J. Chem. Phys. 2018, 148 (24), 241715 10.1063/1.5011181. 29960311

[ref208] Lu C.; Liu Q.; Wang C.; Huang Z.; Lin P.; He L. Molecular Property Prediction: A Multilevel Quantum Interactions Modeling Perspective. Proceedings of the AAAI Conference on Artificial Intelligence 2019, 33, 1052–1060. 10.1609/aaai.v33i01.33011052.

[ref209] Lu J.; Wang C.; Zhang Y. Predicting Molecular Energy Using Force-Field Optimized Geometries and Atomic Vector Representations Learned from an Improved Deep Tensor Neural Network. J. Chem. Theory Comput. 2019, 15 (7), 4113–4121. 10.1021/acs.jctc.9b00001. 31142110 PMC6615995

[ref210] Unke O. T.; Meuwly M. PhysNet: A Neural Network for Predicting Energies, Forces, Dipole Moments, and Partial Charges. J. Chem. Theory Comput. 2019, 15 (6), 3678–3693. 10.1021/acs.jctc.9b00181. 31042390

[ref211] Gasteiger J.; Groß J.; Günnemann S. Directional Message Passing for Molecular Graphs. arXiv:2003.03123 2020.

[ref212] Klicpera J.; Giri S.; Margraf J. T.; Günnemann S. Fast and Uncertainty-Aware Directional Message Passing for Non-Equilibrium Molecules. arXiv:2011.14115 2020.

[ref213] Fang Y.; Zhang Q.; Zhang N.; Chen Z.; Zhuang X.; Shao X.; Fan X.; Chen H. Knowledge graph-enhanced molecular contrastive learning with functional prompt. Nature Machine Intelligence 2023, 5 (5), 542–553. 10.1038/s42256-023-00654-0.

[ref214] Ruddigkeit L.; Deursen R. v.; Blum L. C.; Reymond J.-L. Enumeration of 166 Billion Organic Small Molecules in the Chemical Universe Database GDB-17. J. Chem. Inf. Model. 2012, 52 (11), 2864–2875. 10.1021/ci300415d. 23088335

[ref215] Ramakrishnan R.; Dral P. O.; Rupp M.; Lilienfeld O. A. v. Quantum chemistry structures and properties of 134 kilo molecules. Scientific Data 2014, 1 (1), 140022 10.1038/sdata.2014.22. 25977779 PMC4322582

[ref216] Glavatskikh M.; Leguy J.; Hunault G.; Cauchy T.; Mota B. D. Dataset’s chemical diversity limits the generalizability of machine learning predictions. Journal of Cheminformatics 2019, 11 (1), 69 10.1186/s13321-019-0391-2. 33430991 PMC6852905

[ref217] Hohenberg P.; Kohn W. Inhomogeneous Electron Gas. Phys. Rev. 1964, 136 (3B), B864–B871. 10.1103/PhysRev.136.B864.

[ref218] Riniker S.; Landrum G. A. Better Informed Distance Geometry: Using What We Know To Improve Conformation Generation. J. Chem. Inf. Model. 2015, 55 (12), 2562–2574. 10.1021/acs.jcim.5b00654. 26575315

[ref219] RDKit: Open-Source Cheminformatics Software; 2016. http://www.rdkit.org (accessed 2022-06-15).

[ref220] Mobley D. L.; Guthrie J. P. FreeSolv: a database of experimental and calculated hydration free energies, with input files. Journal of Computer-Aided Molecular Design 2014, 28 (7), 711–720. 10.1007/s10822-014-9747-x. 24928188 PMC4113415

[ref221] Beauman J.; Howard P. Physprop database. Syracuse Research, Syracuse, NY, USA, 1995.

[ref222] Fourches D.; Muratov E.; Tropsha A. Trust, But Verify: On the Importance of Chemical Structure Curation in Cheminformatics and QSAR Modeling Research. J. Chem. Inf. Model. 2010, 50 (7), 1189–1204. 10.1021/ci100176x. 20572635 PMC2989419

[ref223] Korshunova M.; Ginsburg B.; Tropsha A.; Isayev O. OpenChem: A Deep Learning Toolkit for Computational Chemistry and Drug Design. J. Chem. Inf. Model. 2021, 61 (1), 7–13. 10.1021/acs.jcim.0c00971. 33393291

[ref224] Jiang D.; Wu Z.; Hsieh C.-Y.; Chen G.; Liao B.; Wang Z.; Shen C.; Cao D.; Wu J.; Hou T. Could graph neural networks learn better molecular representation for drug discovery? A comparison study of descriptor-based and graph-based models. Journal of Cheminformatics 2021, 13 (1), 12 10.1186/s13321-020-00479-8. 33597034 PMC7888189

[ref225] Wu Z.; Ramsundar B.; Feinberg E. N.; Gomes J.; Geniesse C.; Pappu A. S.; Leswing K.; Pande V. MoleculeNet: a benchmark for molecular machine learning. Chemical Science 2018, 9 (2), 513–530. 10.1039/C7SC02664A. 29629118 PMC5868307

[ref226] Duvenaud D. K.; Maclaurin D.; Iparraguirre J.; Bombarell R.; Hirzel T.; Aspuru-Guzik A.; Adams R. P. Convolutional Networks on Graphs for Learning Molecular Fingerprints; 2015.

[ref227] Coley C. W.; Barzilay R.; Green W. H.; Jaakkola T. S.; Jensen K. F. Convolutional Embedding of Attributed Molecular Graphs for Physical Property Prediction. J. Chem. Inf. Model. 2017, 57 (8), 1757–1772. 10.1021/acs.jcim.6b00601. 28696688

[ref228] Xiong Z.; Wang D.; Liu X.; Zhong F.; Wan X.; Li X.; Li Z.; Luo X.; Chen K.; Jiang H.; et al. Pushing the Boundaries of Molecular Representation for Drug Discovery with the Graph Attention Mechanism. J. Med. Chem. 2020, 63 (16), 8749–8760. 10.1021/acs.jmedchem.9b00959. 31408336

[ref229] Yang K.; Swanson K.; Jin W.; Coley C.; Eiden P.; Gao H.; Guzman-Perez A.; Hopper T.; Kelley B.; Mathea M.; et al. Analyzing Learned Molecular Representations for Property Prediction. J. Chem. Inf. Model. 2019, 59 (8), 3370–3388. 10.1021/acs.jcim.9b00237. 31361484 PMC6727618

[ref230] Pathak Y.; Laghuvarapu S.; Mehta S.; Priyakumar U. D. Chemically Interpretable Graph Interaction Network for Prediction of Pharmacokinetic Properties of Drug-Like Molecules. Proceedings of the AAAI Conference on Artificial Intelligence 2020, 34 (01), 873–880. (acccessed 2023/06/21) 10.1609/aaai.v34i01.5433.

[ref231] Vermeire F. H.; Green W. H. Transfer learning for solvation free energies: From quantum chemistry to experiments. Chemical Engineering Journal 2021, 418, 129307 10.1016/j.cej.2021.129307.

[ref232] Xu K.; Hu W.; Leskovec J.; Jegelka S. How powerful are graph neural networks? arXiv preprint arXiv:1810.00826 2018

[ref233] Dehmamy N.; Barabási A.-L.; Yu R. Understanding the representation power of graph neural networks in learning graph topology. Advances in Neural Information Processing Systems 2019, 32.

[ref234] Garg V.; Jegelka S.; Jaakkola T. Generalization and representational limits of graph neural networks. In International Conference on Machine Learning, 2020; PMLR: pp 3419–3430.

[ref235] Sato R. A survey on the expressive power of graph neural networks. arXiv preprint arXiv:2003.04078 2020.

[ref236] Corso G.; Cavalleri L.; Beaini D.; Liò P.; Veličković P. Principal neighbourhood aggregation for graph nets. Advances in Neural Information Processing Systems 2020, 33, 13260–13271.

[ref237] Schultz H. P. Topological organic chemistry. polyhedranes and prismanes. Journal of Organic Chemistry 1965, 30 (5), 1361–1364. 10.1021/jo01016a005.

[ref238] Paquette L. A.; Ternansky R. J.; Balogh D. W.; Kentgen G. Total synthesis of dodecahedrane. J. Am. Chem. Soc. 1983, 105 (16), 5446–5450. 10.1021/ja00354a043.

[ref239] Cho H.; Choi I. S. Enhanced Deep-Learning Prediction of Molecular Properties via Augmentation of Bond Topology. ChemMedChem. 2019, 14 (17), 1604–1609. 10.1002/cmdc.201900458. 31389167

[ref240] Mater A. C.; Coote M. L. Deep Learning in Chemistry. J. Chem. Inf. Model. 2019, 59 (6), 2545–2559. 10.1021/acs.jcim.9b00266. 31194543

[ref241] Smith J. S.; Isayev O.; Roitberg A. E. ANI-1, A data set of 20 million calculated off-equilibrium conformations for organic molecules. Scientific Data 2017, 4 (1), 170193 10.1038/sdata.2017.193. 29257127 PMC5735918

[ref242] Marenich A. V.; Kelly C. P.; Thompson J. D.; Hawkins G. D.; Chambers C. C.; Giesen D. J.; Winget P.; Cramer C. J.; Truhlar D. G. Minnesota solvation database (MNSOL) version 2012. 2020.

[ref243] Cai C.; Wang S.; Xu Y.; Zhang W.; Tang K.; Ouyang Q.; Lai L.; Pei J. Transfer Learning for Drug Discovery. J. Med. Chem. 2020, 63 (16), 8683–8694. 10.1021/acs.jmedchem.9b02147. 32672961

[ref244] Jiang D.; Wu Z.; Hsieh C. Y.; Chen G.; Liao B.; Wang Z.; Shen C.; Cao D.; Wu J.; Hou T. Could graph neural networks learn better molecular representation for drug discovery? A comparison study of descriptor-based and graph-based models. J. Cheminform 2021, 13 (1), 12 10.1186/s13321-020-00479-8. 33597034 PMC7888189

[ref245] Chen D.; Gao K.; Nguyen D. D.; Chen X.; Jiang Y.; Wei G.-W.; Pan F. Algebraic graph-assisted bidirectional transformers for molecular property prediction. Nat. Commun. 2021, 12 (1), 3521 10.1038/s41467-021-23720-w. 34112777 PMC8192505

[ref246] Wu K.; Zhao Z.; Wang R.; Wei G. W. TopP-S: Persistent homology-based multi-task deep neural networks for simultaneous predictions of partition coefficient and aqueous solubility. Journal of computational chemistry 2018, 39 (20), 1444–1454. 10.1002/jcc.25213. 29633287

[ref247] Jiang J.; Wang R.; Wang M.; Gao K.; Nguyen D. D.; Wei G.-W. Boosting Tree-Assisted Multitask Deep Learning for Small Scientific Datasets. J. Chem. Inf. Model. 2020, 60 (3), 1235–1244. 10.1021/acs.jcim.9b01184. 31977216 PMC7350172

[ref248] Klamt A.; Eckert F.; Arlt W. COSMO-RS: An Alternative to Simulation for Calculating Thermodynamic Properties of Liquid Mixtures. Annu. Rev. Chem. Biomol. Eng. 2010, 1 (1), 101–122. 10.1146/annurev-chembioeng-073009-100903. 22432575

[ref249] Warnau J.; Wichmann K.; Reinisch J. COSMO-RS predictions of logP in the SAMPL7 blind challenge. J. Comput. Aid Mol. Des 2021, 35 (7), 813–818. 10.1007/s10822-021-00395-5. 34125358

[ref250] Kundi V.; Ho J. Predicting Octanol-Water Partition Coefficients: Are Quantum Mechanical Implicit Solvent Models Better than Empirical Fragment-Based Methods?. J. Phys. Chem. B 2019, 123 (31), 6810–6822. 10.1021/acs.jpcb.9b04061. 31343883

[ref251] Bannan C. C.; Calabró G.; Kyu D. Y.; Mobley D. L. Calculating Partition Coefficients of Small Molecules in Octanol/Water and Cyclohexane/Water. J. Chem. Theory Comput. 2016, 12 (8), 4015–4024. 10.1021/acs.jctc.6b00449. 27434695 PMC5053177

[ref252] Lu J.; Zhang Y. Unified Deep Learning Model for Multitask Reaction Predictions with Explanation. J. Chem. Inf. Model. 2022, 62 (6), 1376–1387. 10.1021/acs.jcim.1c01467. 35266390 PMC8960360

[ref253] Gilson M. K.; Liu T.; Baitaluk M.; Nicola G.; Hwang L.; Chong J. BindingDB in 2015: a public database for medicinal chemistry, computational chemistry and systems pharmacology. Nucleic acids research 2016, 44 (D1), D1045–D1053. 10.1093/nar/gkv1072. 26481362 PMC4702793

[ref254] Wishart D. S.; Feunang Y. D.; Guo A. C.; Lo E. J.; Marcu A.; Grant J. R.; Sajed T.; Johnson D.; Li C.; Sayeeda Z.; et al. DrugBank 5.0: a major update to the DrugBank database for 2018. Nucleic Acids Res. 2018, 46 (D1), D1074–d1082. 10.1093/nar/gkx1037. 29126136 PMC5753335

[ref255] Eguida M.; Rognan D. Estimating the Similarity between Protein Pockets. International Journal of Molecular Sciences 2022, 23 (20), 12462 10.3390/ijms232012462. 36293316 PMC9604425

[ref256] Scantlebury J.; Brown N.; Von Delft F.; Deane C. M. Data Set Augmentation Allows Deep Learning-Based Virtual Screening to Better Generalize to Unseen Target Classes and Highlight Important Binding Interactions. J. Chem. Inf. Model. 2020, 60 (8), 3722–3730. 10.1021/acs.jcim.0c00263. 32701288 PMC7611237

[ref257] Zhang X.; Shen C.; Liao B.; Jiang D.; Wang J.; Wu Z.; Du H.; Wang T.; Huo W.; Xu L.; et al. TocoDecoy: A New Approach to Design Unbiased Datasets for Training and Benchmarking Machine-Learning Scoring Functions. J. Med. Chem. 2022, 65 (11), 7918–7932. 10.1021/acs.jmedchem.2c00460. 35642777

[ref258] van Tilborg D.; Alenicheva A.; Grisoni F. Exposing the Limitations of Molecular Machine Learning with Activity Cliffs. J. Chem. Inf. Model. 2022, 62 (23), 5938–5951. 10.1021/acs.jcim.2c01073. 36456532 PMC9749029

[ref259] Xia J.; Tilahun E. L.; Reid T.-E.; Zhang L.; Wang X. S. Benchmarking methods and data sets for ligand enrichment assessment in virtual screening. Methods 2015, 71, 146–157. 10.1016/j.ymeth.2014.11.015. 25481478 PMC4278665

[ref260] Réau M.; Langenfeld F.; Zagury J.-F.; Lagarde N.; Montes M. Decoys Selection in Benchmarking Datasets: Overview and Perspectives. Frontiers in Pharmacology 2018, 9, Review. 10.3389/fphar.2018.00011 PMC5787549 29416509

[ref261] López-López E.; Fernández-de Gortari E.; Medina-Franco J. L. Yes SIR! On the structure-inactivity relationships in drug discovery. Drug Discovery Today 2022, 27 (8), 2353–2362. 10.1016/j.drudis.2022.05.005. 35561964

[ref262] Sundar V.; Colwell L. The Effect of Debiasing Protein-Ligand Binding Data on Generalization. J. Chem. Inf. Model. 2020, 60 (1), 56–62. 10.1021/acs.jcim.9b00415. 31825609

[ref263] Wang L.; Shi S.-H.; Li H.; Zeng X.-X.; Liu S.-Y.; Liu Z.-Q.; Deng Y.-F.; Lu A.-P.; Hou T.-J.; Cao D.-S. Reducing false positive rate of docking-based virtual screening by active learning. Briefings in Bioinformatics 2023, 24 (1), bbac626 (acccessed 7/22/2023) 10.1093/bib/bbac626. 36642412

[ref264] Adeshina Y. O.; Deeds E. J.; Karanicolas J. Machine learning classification can reduce false positives in structure-based virtual screening. Proc. Natl. Acad. Sci. U. S. A. 2020, 117 (31), 18477–18488. (acccessed 2023/07/22) 10.1073/pnas.2000585117. 32669436 PMC7414157

[ref265] Shim H.; Kim H.; Allen J. E.; Wulff H. Pose Classification Using Three-Dimensional Atomic Structure-Based Neural Networks Applied to Ion Channel-Ligand Docking. J. Chem. Inf. Model. 2022, 62 (10), 2301–2315. 10.1021/acs.jcim.1c01510. 35447030 PMC9131459

[ref266] Sieg J.; Flachsenberg F.; Rarey M. In Need of Bias Control: Evaluating Chemical Data for Machine Learning in Structure-Based Virtual Screening. J. Chem. Inf. Model. 2019, 59 (3), 947–961. 10.1021/acs.jcim.8b00712. 30835112

[ref267] Cimermancic P.; Weinkam P.; Rettenmaier T. J.; Bichmann L.; Keedy D. A.; Woldeyes R. A.; Schneidman-Duhovny D.; Demerdash O. N.; Mitchell J. C.; Wells J. A.; et al. CryptoSite: Expanding the Druggable Proteome by Characterization and Prediction of Cryptic Binding Sites. J. Mol. Biol. 2016, 428 (4), 709–719. 10.1016/j.jmb.2016.01.029. 26854760 PMC4794384

[ref268] Meller A.; Ward M.; Borowsky J.; Kshirsagar M.; Lotthammer J. M.; Oviedo F.; Ferres J. L.; Bowman G. R. Predicting locations of cryptic pockets from single protein structures using the PocketMiner graph neural network. Nat. Commun. 2023, 14 (1), 1177 10.1038/s41467-023-36699-3. 36859488 PMC9977097

[ref269] Feixas F.; Lindert S.; Sinko W.; McCammon J. A. Exploring the role of receptor flexibility in structure-based drug discovery. Biophys. Chem. 2014, 186, 31–45. 10.1016/j.bpc.2013.10.007. 24332165 PMC4459653

[ref270] Ravindranath P. A.; Forli S.; Goodsell D. S.; Olson A. J.; Sanner M. F. AutoDockFR: Advances in Protein-Ligand Docking with Explicitly Specified Binding Site Flexibility. PLOS Computational Biology 2015, 11 (12), e1004586 10.1371/journal.pcbi.1004586. 26629955 PMC4667975

[ref271] Guterres H.; Im W. Improving Protein-Ligand Docking Results with High-Throughput Molecular Dynamics Simulations. J. Chem. Inf. Model. 2020, 60 (4), 2189–2198. 10.1021/acs.jcim.0c00057. 32227880 PMC7534544

[ref272] Min Y.; Wei Y.; Wang P.; Wang X.; Li H.; Wu N.; Bauer S.; Zheng S.; Shi Y.; Wang Y.; et al. From Static to Dynamic Structures: Improving Binding Affinity Prediction with a Graph-Based Deep Learning Model. arXiv e-prints 2022, 10.48550/arXiv.2208.10230. PMC11516055 39206846

[ref273] Amaro R. E.; Baudry J.; Chodera J.; Demir Ö.; McCammon J. A.; Miao Y.; Smith J. C. Ensemble Docking in Drug Discovery. Biophys. J. 2018, 114 (10), 2271–2278. 10.1016/j.bpj.2018.02.038. 29606412 PMC6129458

[ref274] Wong C. F. Flexible receptor docking for drug discovery. Expert Opinion on Drug Discovery 2015, 10 (11), 1189–1200. 10.1517/17460441.2015.1078308. 26313123

[ref275] Chandak T.; Mayginnes J. P.; Mayes H.; Wong C. F. Using machine learning to improve ensemble docking for drug discovery. Proteins: Struct., Funct., Bioinf. 2020, 88 (10), 1263–1270. (acccessed 2023/07/24) 10.1002/prot.25899. PMC7815257 32401384

[ref276] Ricci-Lopez J.; Aguila S. A.; Gilson M. K.; Brizuela C. A. Improving Structure-Based Virtual Screening with Ensemble Docking and Machine Learning. J. Chem. Inf. Model. 2021, 61 (11), 5362–5376. 10.1021/acs.jcim.1c00511. 34652141 PMC8865842

[ref277] Mohammadi S.; Narimani Z.; Ashouri M.; Firouzi R.; Karimi-Jafari M. H. Ensemble learning from ensemble docking: revisiting the optimum ensemble size problem. Sci. Rep 2022, 12 (1), 410 10.1038/s41598-021-04448-5. 35013496 PMC8748946

